# Impact of hypersexuality on spousal carers of patients with Parkinson’s disease and frontotemporal dementia: a qualitative study

**DOI:** 10.1136/bmjopen-2024-090870

**Published:** 2025-04-10

**Authors:** Natalie Tayim, Jalesh Panicker, Jennifer Foley, Caroline Selai, Walaa G El Sheikh

**Affiliations:** 1 Program of Clinical Psychology, School of Social Sciences and Humanities, Doha Institute for Graduate Studies, Doha, Ad-Dawhah, Qatar; 2 Department of Uro-Neurology, University College London Hospitals NHS Foundation Trust, London, UK; 3 Department of Brain Repair and Rehabilitation, University College London, London, UK; 4 Department of Neuropsychology, University College London Hospitals NHS Foundation Trust, National Hospital for Neurology and Neurosurgery, London, UK; 5 Department of Clinical and Movement Neurosciences, University College London, London, UK; 6 Faculty of Medicine, American University of Beirut, Beirut, Lebanon

**Keywords:** Caregivers, Parkinson-s disease, Dementia

## Abstract

**Abstract:**

**Objectives:**

Hypersexuality involves an inability to control intense, recurring sexual impulses, resulting in repetitive sexual behaviours. It frequently manifests in patients with neurodegenerative disorders such as Parkinson’s disease (PD) and dementia. Using a qualitative approach, this study aims to explore the impact of hypersexuality on spousal carers of patients with PD and dementia.

**Design:**

Qualitative study using semistructured interviews and thematic analysis.

**Setting:**

This study was conducted in secondary care settings, including movement disorder and dementia clinics, as well as through patient support organisations. Participants were recruited from multiple centres across the UK. Interviews were conducted in a clinical research setting.

**Participants:**

Eight spousal carers (five caring for patients with PD, three for patients with dementia) participated in the study. Participants were selected based on their role as primary carers and their experience managing hypersexuality in their partners.

**Results:**

The thematic analysis identified 12 themes: manifestations, sexual practices, impact, control, emotional formulations, beliefs in causes of hypersexuality and attributions, relationship with the partner, dealing with hypersexuality, coping with hypersexuality, self-image, stigma and professional help-seeking. Hypersexuality altered patients’ sexual cognitions and behaviours, causing distress and strain on carers’ mental health and marital life. Carers struggled to cope with their partners’ hypersexuality, facing emotional burden and barriers to seeking professional help.

**Conclusions:**

Hypersexuality significantly impacts spousal carers of patients with PD and dementia, affecting their emotional well-being and relationships. Healthcare professionals should recognise and address hypersexuality’s psychological and relational consequences. Psychoeducation, support groups and tailored interventions for patients and carers are recommended to alleviate emotional distress. Future research should explore the broader familial impact of hypersexuality and develop effective management strategies.

**STRENGTHS AND LIMITATIONS OF THIS STUDY**

- This study provides qualitative insights into the experiences of spousal carers managing hypersexuality in patients with PD and dementia.
- The use of semistructured interviews allows for an in-depth exploration of carer perspectives.
- Potential under-reporting of hypersexuality due to stigma may have influenced the data.
- The study focuses solely on spousal carers, excluding experiences of other family members or care professionals.

## Introduction

 Hypersexuality, classified under compulsive sexual behaviour disorder in the International Classification of Diseases (ICD) 11th Revision, involves an inability to control intense, recurring sexual impulses, resulting in repetitive sexual behaviours, which can lead to distress and impairment in personal, social or occupational functioning.^1^ Hypersexuality frequently manifests as a disorder in patients with neurodegenerative disorders such as Parkinson’s disease (PD) and dementia.^2^ It typically arises as a side effect of dopamine replacement therapy in PD^3^ and as a result of frontal lesions in dementia.^4^ The management of hypersexuality often involves the reduction or cessation of behaviour-inducing drugs in PD and a switch to alternative medications like levodopa, catechol-O-methyltransferase inhibitors or monoamine oxidase B inhibitors.^5 6^

As patients get older, they tend to become increasingly dependent on family members for support.^7^ Accumulating responsibilities on the carer can lead to carer burden, which encompasses a range of negative responses such as a decrease in quality of life and physical and psychological deterioration.^8^ For example, spouses and female carers of patients with frontotemporal dementia (FTD) tend to experience distress, increased rates of depression and poor sleep.^9^ Hypersexuality can worsen carer burden and be detrimental to the patients’ and their partners’ quality of life.^10 11^ Accounts of spousal carers of patients with neurological disorders suffering from hypersexuality are lacking in the literature. Therefore, using a qualitative approach, the current study aims to explore the impact of hypersexuality on spousal carers of patients with PD and dementia.

## Methods

### Ethics

This study (ethics application ID: 15/LO/0557) was approved by the London-Hampstead National Research Ethics Committee.

### Study design

This study employed a phenomenological qualitative approach. This approach was deemed the most appropriate for the present study since the intention of this study is to understand the spousal carers’ personal experiences of the phenomenon of hypersexuality and how they view and interpret their experiences.

This study was conducted from April 2015 to August 2017. It was part of a broader University College London (UCL) project examining hypersexuality in neurological disorders.^12 13^

### Eligibility criteria

Carers were included in the study if they are spouses or partners of patients with clinically diagnosed PD according to the UK Brain Bank Criteria or clinically diagnosed FTD, indicated hypersexuality either in the past or present since developing PD or dementia and have the ability to provide informed consent.

Carers were excluded from the study if they were spouses or partners of patients with hypersexuality predating the onset of PD or FTD, the patients had coexisting neurological disorders as determined by clinical history or they had difficulty understanding/speaking English.

### Measure

#### Carer assessment interview

The interview is a semistructured 34-item interview, developed by NT. During the interviews, the participants were asked to reflect on, describe and/or recount their experience with hypersexuality and its impact on their lives to the best of their abilities considering the sensitive nature of the topic (online supplemental file 1).

### Procedure

Spouses of patients with PD who indicated hypersexuality as being an issue during patients’ clinical appointments and who were prepared to discuss it in further detail with a researcher were contacted by NT. These carers, as well as the carers who contacted the researchers after reading information leaflets about the study circulated by Parkinson’s UK, were further informed about the study’s aims, methods, potential risks and benefits and confidentiality over the phone.

Carers of patients with FTD or Alzheimer’s disease (AD) were informed about the study by the clinical staff at the Dementia Research Centre (DRC), through either the newsletter that was sent out periodically which contained blurbs about the study and the contact details of the members of the research team or through the carer leaflets passed out at the Frontotemporal Dementia Support Group 5 March 2016 Seminar, which took place at 33 Queen Square. These carers were further informed about the study’s aims, methods, potential risks and benefits and confidentiality over the phone, as well as that the interview portion of the study was going to be audio-recorded using a Dictaphone and that the recorded material was only to be used in writing up the transcripts, which was completed almost immediately after assessment. Participants were assured that the recorded material would not be passed on and that it would be deleted at the end of transcription. Participants who did not consent to the use of the Dictaphone were informed that they were still eligible to take part in the study.

Interested carers were then asked to come into the Department of Uro-Neurology at the National Hospital for Neurology and Neurosurgery (NHNN) where any of the available rooms on the scheduled dates was used to provide the participants with written information about the study, obtain written consent and consequently complete assessment.

**Figure 1:**
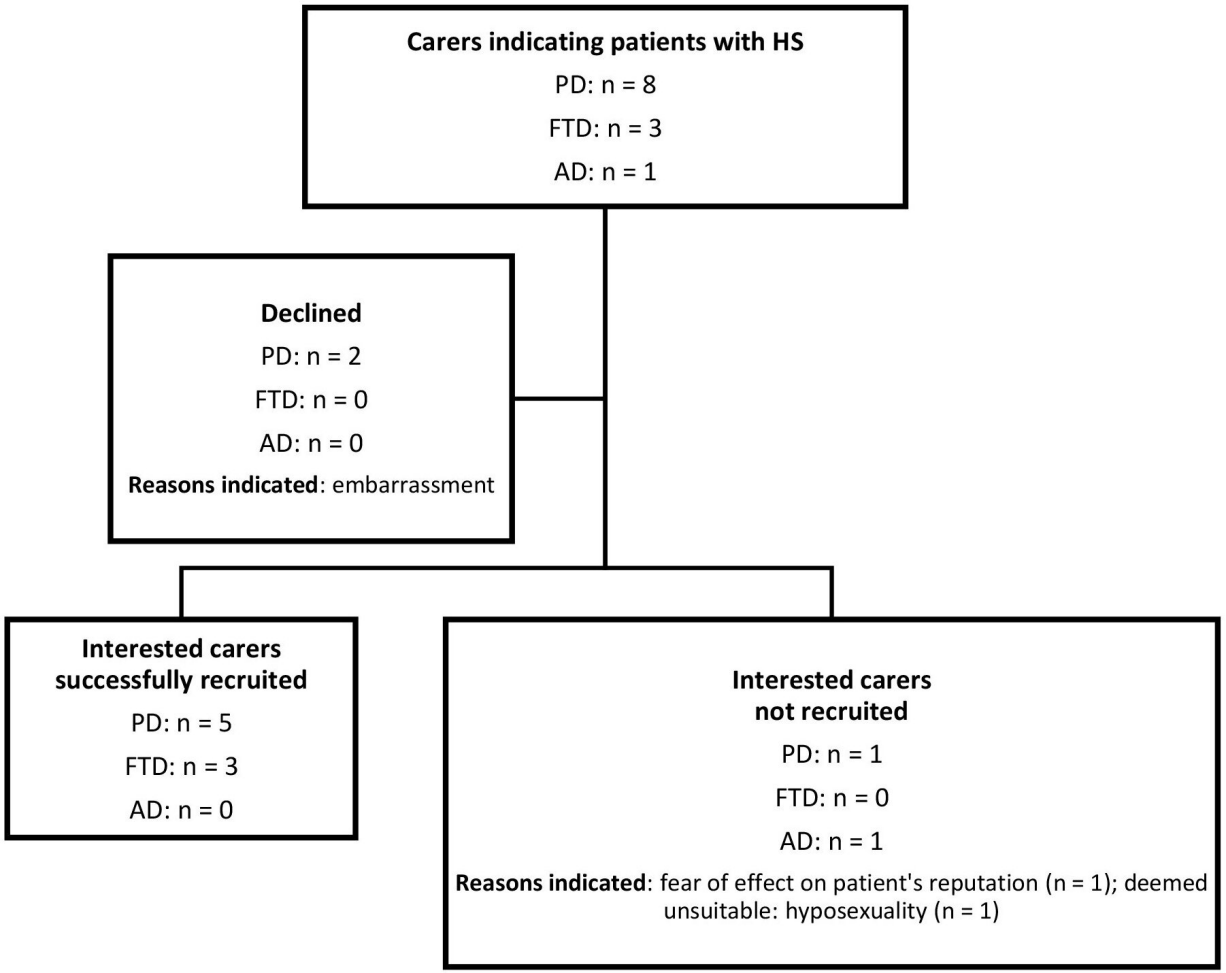
Flowchart summarising the recruitment results for Parkinson’s disease and dementia carers. AD, Alzheimer’s disease; FTD, frontotemporal dementia; HS, hypersexuality; PD, Parkinson’s disease.

A total of 12 carers indicated hypersexuality as having been or still being an issue, eight of whom were carers of patients with PD, four of whom were carers of patients with FTD and none of whom were carers of patients with AD. Eight carers were successfully recruited into the study. Five PD carers were recruited from the Movement Disorders Centre at the NHNN, Edgware Community Hospital and from Parkinson’s UK. Three FTD carers were recruited from the DRC at the NHNN. Figure 1 presents a summary of recruitment results for PD and dementia carers.

The interviews were conducted by NT, a PhD candidate at the time of the research. As part of her doctoral thesis, she drew on her undergraduate background in psychology to inform her approach to qualitative data collection. NT had no prior relationship with the study participants before the research commenced. Participants were informed about the study’s purpose through a leaflet, which explained the association between neurological disorders and changes in sexual desire, as well as the study’s aims to understand these changes and their impact. The leaflet also provided details about the study’s collaboration between the DRC and the Department of Uroneurology at Queen Square, emphasising the potential benefits of the research in improving care and developing psychological interventions. Additional details regarding the interviewer’s background and role are available in the study materials provided to participants. The interviews ranged from 2 hours to nearly 4 hours in duration, with as many breaks as required by the participants.

### Sample size

The sample for this qualitative study comprised eight carers, a size considered sufficient for exploratory research within qualitative methodologies. Qualitative research prioritises in-depth understanding over statistical generalisability, with the sample size determined by the principle of thematic saturation. Saturation, in this context, refers to the point where additional data collection yields no new insights relevant to the research questions.^14 15^ This approach aligns with that of Fusch and Ness, who emphasise that ‘more is not necessarily better than less,’ challenging the notion of a fixed target number for saturation.^15^ Instead, saturation is reached when the data adequately represent the phenomenon under study and enable study replication, and further coding produces redundant information. Moreover, Guest *et al* (2006) posit that a sample of six can generate ‘basic elements for metathemes’, especially in studies involving sensitive topics.^16^ Consequently, the data obtained from eight carers allowed for a thorough exploration of individual experiences, contributing to theory development within the inherent constraints of qualitative research.

### Patient and public involvement

It was not appropriate or possible to involve patients or the public in the design, conduct, reporting or dissemination plans of our research.

### Data analysis

Braun and Clarke’s thematic analysis approach was used to analyse the qualitative data for this study.^17^ We adhered to the thematic analysis process, which included becoming familiar with the data, organising the data, generating initial codes, generating themes, naming and defining themes, producing the report and determining the quality of analysis.

Initially, interview transcripts were reviewed and organised into an Excel chart to facilitate data accessibility and ensure comprehensive analysis. This systematic arrangement allowed researchers to examine participant responses to each interview question without repeatedly referring to full transcripts. Following data familiarisation, key extracts were identified through annotation and highlighting, capturing recurring words, ideas and patterns. These extracts were systematically grouped into codes by NT and the study supervisors. Researchers then compared and refined codes through discussion, establishing coherent relationships and categorising them into preliminary themes. Themes were subsequently reviewed for coherence, consistency and distinctiveness. Based on this evaluation, themes were retained, modified or removed as necessary. Subthemes were identified where applicable, representing distinct yet interconnected elements within overarching themes.

Finally, the thematic analysis was checked against a 15-point checklist of criteria for good thematic analysis, which was produced by Braun and Clarke (p96).^17^

### Rigour and reflexivity

To ensure methodological rigour, we adhered to the Consolidated Criteria for Reporting Qualitative Research.^18^ Strategies to enhance trustworthiness included investigator triangulation, whereby multiple researchers participated in coding, theme generation and data interpretation to minimise individual biases. Member checking was conducted informally, allowing participants to clarify or expand on their responses during interviews, ensuring the authenticity of the data. Reflexivity was maintained throughout the research process, with researchers critically examining their own preconceptions and potential influences on data collection and analysis. Regular discussions within the research team facilitated awareness of positionality and its impact on interpretation, thereby strengthening the credibility and dependability of the findings.

## Results

### Characteristics of the sample

A total of n=8 carers (PD: n=5 and FTD: n=3) agreed to participate in this study. Table 1 summarises the descriptive characteristics of the carer sample.

**Table 1:** Carer sample descriptives

Variable	Carer 1	Carer 2	Carer 3	Carer 4	Carer 5	Carer 6	Carer 7	Carer 8
Neurological disorder	PD	PD	PD	PD	PD	FTD	FTD	FTD
Medications at the time of hypersexuality*†	Stalevo Rasagiline Clonazepam Fludrocortisone Movicol Atropine	Ropinirole Amantadine Selegiline Madopar Stalevo	Ropinirole Madopar Citalopram	Ropinirole Rasagiline Entacapone Amantadine	Ropinirole Madopar Stalevo	–	–	–
Implicating medications*†	Stalevo	Ropinirole	Unsure (Ropinirole)	Rasagiline	Ropinirole	–	–	–
Implicating medication reduced or discontinued*†	Yes discontinued	Yes discontinued	Yes discontinued	Yes discontinued	No	–	–	–
Still hypersexual†	Deceased	Yes	Yes	No	Yes	Deceased	Yes	Yes
DBS*	No	Yes	No	No	No	–	–	–
Type	–	Bilateral STN	–	–	–	–	–	–
Associated symptoms								
Sexual behaviour	Preoccupation with sexIncreased desire for sex generallyChange in sexual orientationUncontrollable masturbationPornographySex phone linesSex channelsVisiting sex shops	Preoccupation with sexIncreased desire for sex with wife and generallyHaving sex more frequentlyIncreased masturbationPornographyFetishism	Preoccupation with sexIncreased desire for sex with husband and generallyHaving sex more frequentlyInsatiable desire for masturbation	Increased desire for sex with husbandHaving sex more frequentlySexual attraction for therapistHaving sex on stairsHint of S&MPornography	Preoccupation with sexIncreased desire for sex with wife and generallyIncreased masturbationPornographySex phone lineDating sites	Preoccupation with sexIncreased desire for sex generallyPornographySex phone linesDating sitesMassage parloursProstitutes	Preoccupation with sexIncreased desire for sex with wife and generallyHaving sex more frequentlyIncreased masturbation	Preoccupation with sexIncreased desire for sex generallyIncreased masturbationPornographyDeviant interestsFantasies of dressing in women’s underwear
Other impulse control disorders	None	Compulsive eatingCompulsive buying	None	Compulsive eating	None	Compulsive buying	Compulsive eating	Compulsive eatingCompulsive buying
Other compulsive behaviours	None	None	None	Desire to move	None	None	None	Clock-watchingWriting down electricity and water readings

* There isare no data available for the respective variables for Carers 5, 6, and 7, criteria only applicable to PD patients.Information obtained from s clinical notes.

† Information obtained from patient’s clinical notes.

DBS, deep brain stimulation; FTD, frontotemporal dementia; PD, Parkinson’s disease; STN, subthalamic nucleus.

### Qualitative thematic analysis

**Figure 2:**
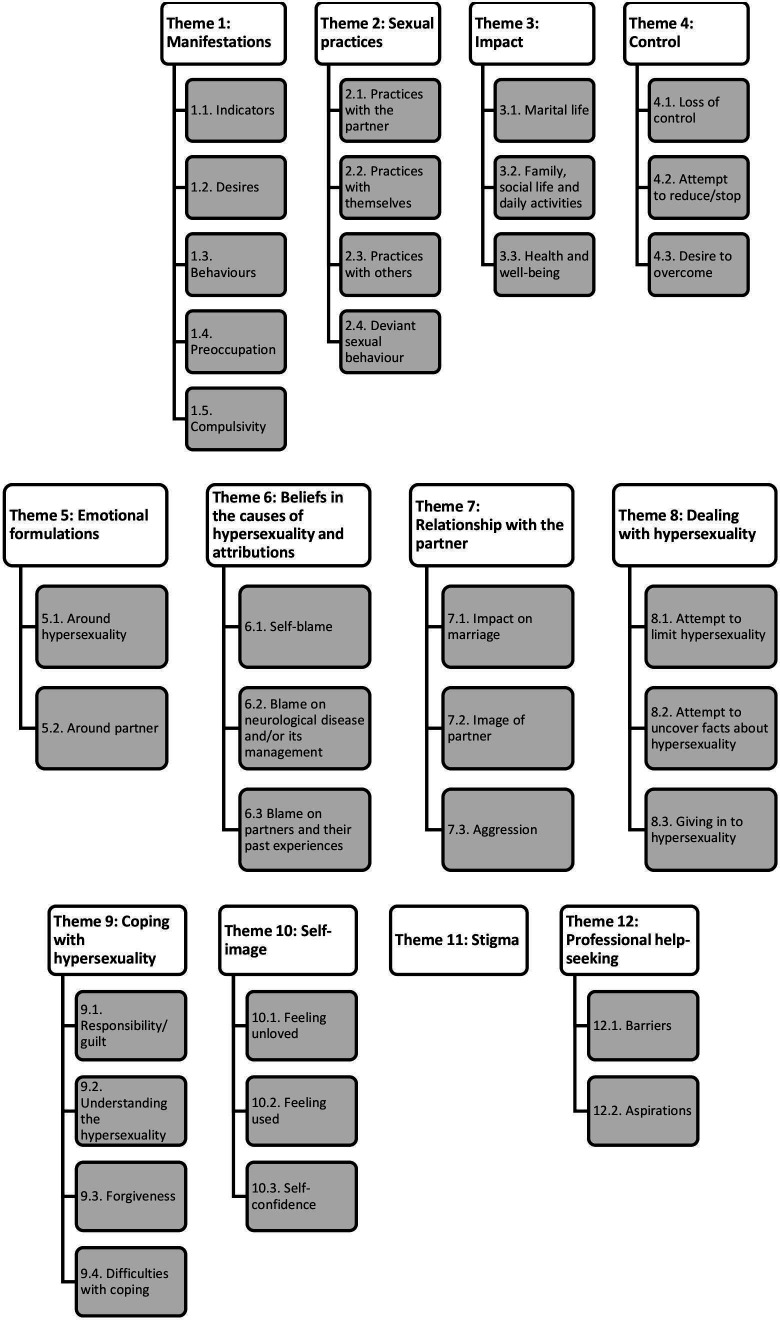
Themes and subthemes identified in the interviews.

Twelve themes emerged from the interview data of PD and FTD carers and are as follows: manifestations, sexual practices, impact, control, emotional formulations, beliefs in causes of hypersexuality and attributions, relationship with the partner, dealing with hypersexuality, coping with hypersexuality, self-image, stigma and professional help-seeking (figure 2).

Quotes under each theme are presented in online supplemental table S1.

### Theme 1: Manifestations

This theme outlines the carer-perceived manifestations of hypersexuality in their partners, encompassing five identified subthemes.

#### Indicators

The carers provided accounts of how they became cognizant of the hypersexuality. These instances, termed ‘indicators’, fell broadly into three categories: (1) their partners told them directly about their hypersexuality, (2) they found out based on changes in their partners’ sexual behaviours towards them or (3) they discovered their partners’ clandestine behaviours (Carer 1).

I found a till receipt for a gay magazine… I sat on the knowledge for a couple of weeks but first of all I went straight up to WH Smith and bought a copy of the magazine thinking that either it wasn’t what I thought it was… it was Gay Times… or this had been bought by mistake… I got a copy… I sat there outside and read it and realised it was highly unlikely that it had been bought by mistake… (Carer 1)

#### Desires

Increased desire following the onset of hypersexuality was evident in carers’ accounts. The predominant response involved partners exhibiting heightened desire in sexual activity within and outside the relationship, as well as engaging in self-pleasure through masturbation and the use of pornographic material (Carer 7).

That’s the only thing he’s interested in … to have sex… (Carer 7)

#### Behaviours

Furthermore, the hypersexuality apparently caused changes in pre-existing behaviour or the development of new behaviours. These changes fell broadly into two categories: (1) the adoption of pornographic materials or new sexual behaviours involving others and (2) an increase in the levels or forms of sexual behaviours towards partners or the intensification of old sexual behaviours (Carer 4).

Normally she likes tenderness and sweetness and this was sort of a bit more lust… go for it… behavior was extreme if you like because she’s a reserved person … who has other high standards of good behavior… so this was like nature in the raw really… (Carer 4).

#### Preoccupation

One of the main manifestations of hypersexuality was preoccupation with sexual thoughts (Carer 3).

her thoughts are uncontrollable and come so much of the time… (Carer 3)

#### Compulsivity

Carers perceived that their partners’ preoccupation with sexual thoughts translated into compulsive behaviour, another main manifestation of hypersexuality. Reported compulsive behaviours varied and encompassed frequent or intense consumption of pornographic materials, visiting prostitutes and generally indulging in sexual behaviours throughout the day (Carer 3).

Hypersexuality is present all throughout the day and during the night while I am asleep… (Carer 3)

### Theme 2: Sexual practices

This theme outlines the carer-perceived impacts of hypersexuality on their partners’ sexual practices, encompassing four identified subthemes.

#### Practices with the partner

Sexual practices with the partner underwent changes in both the frequency and nature of sexual acts. Certain carers reported that their partners, upon developing hypersexuality, expressed an increased demand for sexual activity with them (Carer 7).

And now [he was asking for sex] every morning… every evening… sometimes he’s asking during the day… (Carer 7)

Additionally, a majority of carers noted changes in the nature of their partners’ sexual demands or behaviours, often describing them as being out of character with the person they were before developing hypersexuality. These changes included, for instance, more aggressive sexual tendencies, demands for role play and a shift towards more adventurous sexual practices, such as oral or anal sex, which deviated from their previous patterns (Carer 4).

She didn’t ask for Fifty Shades of Grey no… but still … a little hint of S&M which really wasn’t part of our repertoire… (Carer 4)

Moreover, certain carers reported a decrease in sexual activity with their partner—in some cases because they started to resist their frequent or inappropriate advances. In other cases, the decline in marital sexual activity seemingly occurred as the partner sought gratification from alternative sources.

#### Practices with themselves

The majority of carers reported that their partners also indulged in masturbation and use of pornographic material.

#### Practices with others

Sexual practices with others included anonymous sexual encounters, paying for sex and developing sexual interest in individuals other than the spouse.

#### Deviant sexual behaviour

Lastly, desires did not appear to translate into paraphilic deviant practices as only one carer reported this (Carer 8).

It needs to be more upfront that it’s not just about a decrease in sex or an increase in sex… it could be a decrease in a normal sexual relationship and a… a subverted or a hidden cover increase in some kind of deviant sexual behavior which had been what was going on for twenty years and I didn’t know about… (Carer 8)

**Figure 3:**
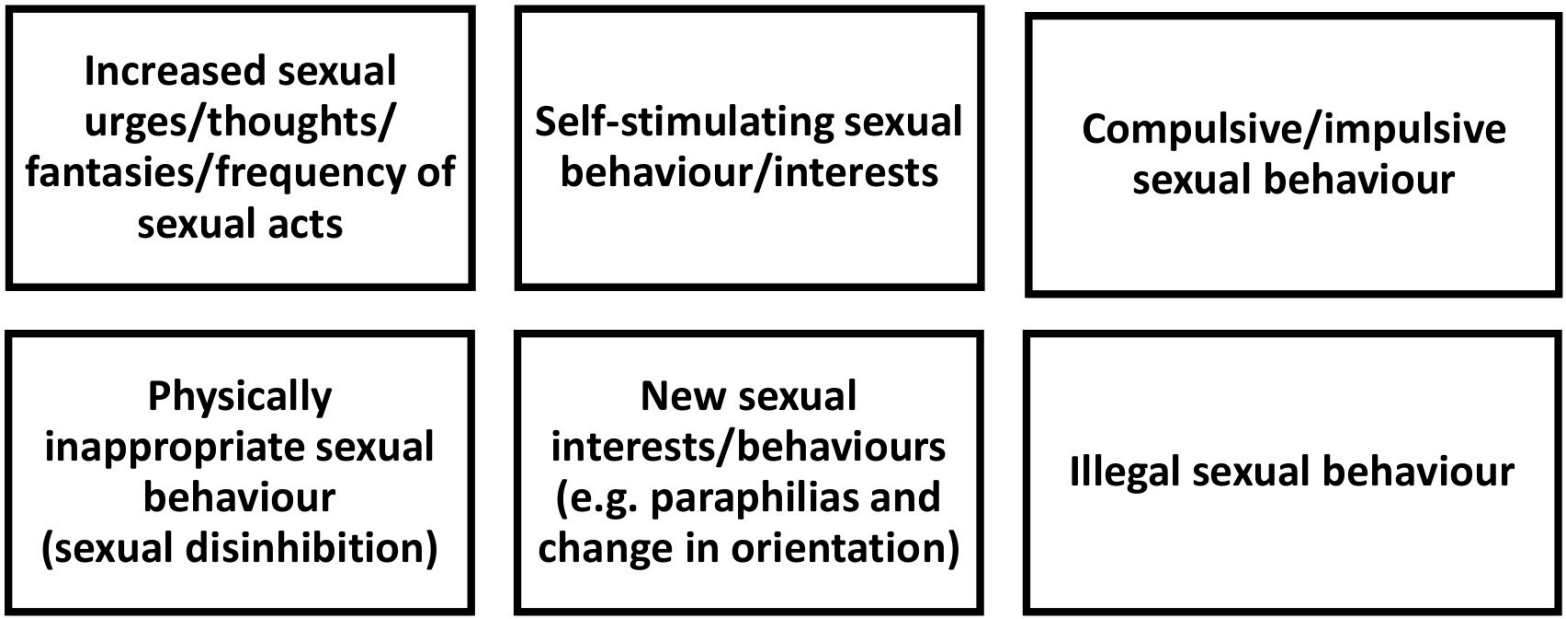
Summary of categories for clinical phenomenology of hypersexuality in patients with Parkinson’s disease and dementia.

Themes 1 and 2 illustrate the clinical phenomenology of hypersexuality. These changes can be summarised using the categories presented in figure 3.

### Theme 3: Impact

This theme outlines the carer-perceived impacts of hypersexuality on their partners’ different areas of daily living, encompassing three identified subthemes.

#### Marital life

Nearly all carers conveyed that hypersexuality had adverse effects on their marital lives, resulting in diminished intimacy, increased emotional distance between themselves and their partner and a spectrum of negative emotions on their part. These included feelings of anger, betrayal, despair, disapproval, embarrassment, reduced self-confidence, sadness and self-blame. Primarily, the impersonal or mechanical nature of their partners’ increased demand for sexual activity had generated feelings of disgust or resentment on the part of their spouses. Additionally, these demands altered the nature of their sexual relationship in ways that were unwelcome to the spouses (Carer 1).

It was dreadful… devastating … I couldn’t make head and the tail of it… it just didn’t add up to the man I’d been living with for nearly fifty years… (Carer 1)

Furthermore, certain responses indicated a significant transformation in the nature of the marital relationship. This shift was characterised by a growing lack of respect for the partner and, in some instances, a perceived need to exert control over them in an effort to preserve the marriage (Carer 8).

I’ve lost respect for him… how can you respect someone that gets off of watching little boys being humiliated… I’ve said to him I won’t collude or condone with anything he’s done… and I won’t accept those things either… and that whilst he lives in the house with me he behaves in a way I would want him to behave legally… (Carer 8)

Many carers emphasised that their partners had become markedly less affectionate and loving towards them in general since the onset of hypersexuality (Carer 1).

I’ve always thought of it very old fashioned as making love… sex for sex’s sake for me is nothing… so the fact that he was then using these magazines to psych himself up to come and have sex with me was really meaning he was just using me to have sex… (Carer 1)

#### Family, social life and daily activities

Half of the carers reported that hypersexuality had a detrimental impact on their family lives, noting effects on their children that ranged from fathers being absent much of the time to children experiencing trauma or stress due to their father’s hypersexuality (Carer 1).

My kids were shocked, so mentally and emotionally distanced themselves… (Carer 1)

Moreover, hypersexuality had a negative impact on the partners’ finances, particularly for those whose hypersexual behaviours involved visits to sex shops for purchases or spending time with prostitutes (Carer 1).

I decided that if he agreed… and he did agree… that I would take his credit and debit cards off him… hide any money I’d got in the house… I left him… I think we agreed on fifteen pounds which would be enough for taxi and whatever so he couldn’t do that… I hid the cheque books and hid any money I’d got in the house so he didn’t have any access to cash… and that worked well for a couple of months and then he remembered that he had an account that I’d forgotten about… (Carer 1)

#### Health and well-being

Half of the carers reported that their partners experienced sleep disturbances, mood deterioration and overall poor mental health, as a result of the hypersexuality (Carer 8).

He seemed very withdrawn… he was completely locked into this mad behavior… there was no happiness… there was no joy… he never smiled… he was apathetic… he was almost irritable… he was quite angry… he doesn’t get irritable… he doesn’t show it… if he is and this is what frightens me about him… I feel it’s like watching a pressure cooker and there’s going to be a time when it pops… (Carer 8)

Concerning the impact of hypersexuality on their partners’ self-confidence, the findings were not clear-cut, with some participants noting a positive and some a negative impact, while others were unsure whether their partners’ self-confidence had been affected at all (Carer 4).

Probably more confident… I mean she was writing at the time… that’s her identity… she’s a writer… (Carer 4)

Regarding the impact of hypersexuality on their partners’ quality of life, the findings were similarly mixed. Four carers mentioned a negative impact, with one providing an explanation. This carer specified that her husband felt he now had a wife who did not love him as much as before, leading to a general sense of deflation.

### Theme 4: Control

This theme outlines the carers’ perceptions regarding how much control they believed their partners had over their hypersexuality. We identified three subthemes.

#### Loss of control

All carers believed their partners lacked control over their sexual behaviour, but the extent of this loss varied across individuals (Carer 1).

He couldn’t resist it… it was hopeless… he couldn’t stop it… (Carer 1)

Carers of patients with dementia characterised them as ‘*disinhibited’* (Carer 8).

There is a difference… the impulse to do something and the ability to know right from wrong… he knows what’s right and what’s wrong but he chose to take a risk and his risk-taking has increased… he is the one with his hand on his penis… (Carer 8)

#### Attempt to reduce/stop

Half of the carers reported that their partners attempted to reduce or stop their hypersexuality, with varying degrees of success reported among them (Carer 2).

I think he’s doing a good job in trying to keep a lid on it… it’s still there but more controlled… (Carer 2)

#### Desire to overcome

More than half of the carers noted that their partners expressed a desire to overcome their hypersexuality. This was either conveyed through direct verbalisation to the carers or others or inferred from observable efforts to control their behaviours, such as reduced requests for sex (Carer 1).

[He] desperately wanted to stop it… he just couldn’t work out what had hit him… (Carer 1)

### Theme 5: Emotional formulations

This theme outlines the emotional formulations that the carers had around their partners and/or around hypersexuality itself.

#### Around hypersexuality

At least half of the carers found hypersexuality to be a perplexing phenomenon, leading to a negative emotional formulation marked by shock, confusion and horror, as they grappled with the profound changes in their long-term partners’ feelings and behaviours (Carer 8).

I just didn’t know what had happened … it’s like waking up on the other side of the mirror like Alice in… Through the Looking Glass… it was just so abnormal… he was cold towards me… (Carer 8)

Other carers expressed more positive emotional formulations around hypersexuality. For instance, one carer conveyed emotions like amusement and interest in response to his wife’s newly developed lustful approach (Carer 4).

Normally she likes tenderness and sweetness and this was sort of a bit more lust… go for it… (laughing)… and in a way that was fresh and amusing… again one took that as a positive thing… for a while anyway… (Carer 4)

#### Around partner

With the exception of one carer, all carers developed negative emotional formulations around their partners due to hypersexuality. These negative emotions encompassed annoyance, betrayal, despair, embarrassment, hurt, irritation, pity and repulsion. These emotions often evolved and changed over time in tandem with the partner’s shifting behaviours (Carer 8).

I was so angry… it wasn’t just emotion… there was anger… I felt very angry about what he’d done … I wouldn’t want him to touch me because I don’t know who he is… he was doing things that are completely unacceptable… sad… I was very sad… I felt rejected… I felt confused… I feel such a fool… let down… (Carer 8)

It is noteworthy that carers found it challenging to separate their emotional formulations around their partners from those around hypersexuality in itself. This may be indicative that the effects of hypersexuality are overwhelming enough to cause the carers to regard them as being one and the same.

### Theme 6: Beliefs in the causes of hypersexuality and attributions

This theme outlines the carers’ opinions about the perceived reasons for the onset and progression of hypersexuality. We identified three subthemes.

#### Self-blame

Certain carers attributed the onset of hypersexuality to themselves (Carer 5).

The longer he’s not having sex the worse it’s making him… so basically that might be my fault… (Carer 5)

#### Blame on neurological disease and/or its management

Attribution of hypersexuality to the neurological disease and/or its management was the main reason given by carers for the development of their partner’s hypersexuality. All five carers of the PD patients attributed the hypersexuality to the PD and its management (pharmacological and surgical) (Carer 5).

I suppose now I can point to Ropinirole and say it’s Ropinirole’s fault… (Carer 5)

The three carers of the FTD patients, on the other hand, attributed the hypersexuality to the FTD as there had been no sign of it before its onset (Carer 6).

I think it just came with the disease… right before he passed I said to him ‘You couldn’t help it… it wasn’t you… it wasn’t what you were like… it was a disease and you’ve got two of them and they’re both serious’… (Carer 6)

#### Blame on partners and their past experiences

Half of the carers attributed at least some aspects of the hypersexuality to their partner’ past experiences (Carer 1).

[Husband’s] parents were away… he was allowed… for a night… and he was allowed to ask his friend from his school to stay overnight which he did… and then some sort of homosexual activity occurred… I mean the implication has always been that he was a repressed homosexual and the hypersexuality had overridden his control of that and was forcing him… allowing him… whatever… stimulating him to pursue the homosexuality as he never had done as far as I know… (Carer 1)

Carer 6 suggested that her husband’s hypersexuality might stem from two previous experiences. First, he had been sexually abused as a 7-year-old child by the headmaster of his school. Second, he had an ex-girlfriend of Indian descent during his twenties who died in a car accident. She indicated that both prostitutes her husband had been involved with were dark-skinned and considered that there might be a link between this and the evolution of his hypersexuality (Carer 6).

### Theme 7: Relationship with the partner

This theme outlines the carer-perceived impacts of hypersexuality on the carers’ relationships with their partners, encompassing three identified subthemes.

#### Impact on marriage

Certain carers highlighted changes in the nature of marital sexual activity, a decrease in affection between partners and a shift in the overall balance of the relationship (Carer 7).

It’s not like an intimate loving relationship… it’s more mechanical and ritual-like… (Carer 7)

#### Image of partner

Some carers stressed that their image of their partners had changed due to their hypersexual behaviours. It seemed that these carers no longer regarded their partners as the same individuals they were before developing hypersexuality, indicating a difficulty in distinguishing between their partners as individuals and the hypersexuality itself (Carer 1).

It just didn’t add up to the man I’d been living with for nearly fifty years… (Carer 1)

#### Aggression

Evidently, certain carers, experiencing stress and frustration from dealing with their partners and their hypersexuality, expressed either a desire or an actual instance of having an aggressive response to their partners’ hypersexuality (Carer 2).

I think the worst thing was that on one occasion I actually momentarily considered violence towards him… he’d had one of his trips to the sex shop… he got stuff… I’d been out in the garden… and I’d seen him through the window of his office… obviously he was busy looking at some stuff… and it was lunch time and I came in to give him his lunch… and I stood behind him and I really can’t believe it now but I’ve got to tell you… it’s the truth… I stood behind him with this big hammer in my hand… and I thought quite clearly ‘A couple of blows to your skull with this and this would all be over’… and then I put the hammer away and served his lunch… (Carer 2)

### Theme 8: Dealing with hypersexuality

This theme outlines the various ways in which the carers dealt with their partners’ hypersexuality, encompassing three identified subthemes.

#### Attempt to limit hypersexuality

Carers attempted to limit hypersexuality by placing blocks on the computer, for instance, so that their partner could no longer access any pornography (Carer 8).

If he did continue to do something and the police came… I would step aside… I would explain that he had a degenerative brain disease but I’m not going to protect him if he’s doing something illegal which he was… I think there’s a limit to how much protection I can afford someone who has done nothing to deserve protection… (Carer 8)

#### Attempt to uncover facts about hypersexuality

Half of the carers reported actively attempting to investigate their partner’s hypersexual behaviours. This included actions such as searching for hidden pornographic materials, checking computers or phones for evidence of visits to sex sites and examining phones for messages from other individuals that they might be involved with sexually (Carer 1).

I certainly looked for materials he’d obtained and was using… when I realised that he had bought a gay magazine… because I found the receipt by chance… after it when I thought about it he just said he was just curious… when he was out I went and unlocked the case and found more magazines… so yes I did go looking for them… yes I did go and look in his case and see what he’d got… (Carer 1)

#### Giving in to hypersexuality

Approximately half of the carers acknowledged their partner’s hypersexual behaviours, although with dissatisfaction. For a small number, this acceptance extended to a greater degree of understanding and even support in helping their partner to indulge their hypersexual desires outside of the marital relationship (Carer 1).

I thought ‘God this poor man has been a repressed gay all his life… he’s never indulged in it… I know he’s ill… he hasn’t got that many more years to live… if he wants to indulge in this why shouldn’t he?’ and so I said to him ‘Look you can’t drive now… if you want to go to gay bars and clubs I will take you there’… after you’d phoned me and said that there is some evidence that it does alter sexual orientation… I just sat and cried… I thought ‘Poor man’… he must’ve been so confused with what’s happening to him… utterly… and he couldn’t resist it… (Carer 1)

### Theme 9: Coping with hypersexuality

This theme outlines the various ways in which the carers coped with their partners’ hypersexuality, encompassing three identified subthemes.

#### Responsibility/guilt

Except for one carer, all indicated no responsibility for their partners’ hypersexuality. This lack of perceived responsibility may aid in maintaining necessary psychological and emotional distance to cope with the situation’s stress and pressure (Carer 8).

I thought I had done something and I tried for twenty years to find out what it was and when I found out it had all been him I didn’t feel responsible… (Carer 8)

#### Understanding hypersexuality

All carers recognised the neurological origin of hypersexuality, yet this understanding did not uniformly translate into effective coping. Certain carers exhibited a more nuanced comprehension of the condition and its manifestations (Carer 7).

Kind of owning the fact that… that sex is not just with the other… it’s your relationship with yourself as well as the other person so I’m able to separate how to be who I am and who he is so I don’t actually feel exploited… like I’m able just to see that he has a greater need for sex than me and for our relationship to work I help him to meet that need and I’m having lots of other needs met in our relationship… it balances quite nicely… (Carer 7)

#### Forgiveness

Certain carers could forgive their partners for their hypersexuality, while others saw no need for forgiveness. Those considering forgiveness found it challenging and could only achieve it sometime in the future. Carer 1, for example, reported that she was ‘*on the road to forgiveness’*.

#### Difficulties with coping

Coping with hypersexuality is challenging, with around half of carers facing difficulties, and for a few, leading to a desire to no longer exist (Carer 8).

[I] didn’t want to commit suicide but I would like not to exist and there’s a difference between not wanting to exist and wanting to be dead… (Carer 8)

### Theme 10: Self-image

This theme outlines the carer-perceived effects of hypersexuality on the carers’ self-image, encompassing three identified subthemes.

#### Feeling unloved

Half of the carers felt unloved by their husbands, especially when the sexual relationship became mechanical and non-affectionate due to hypersexuality. This evoked sadness and nostalgia for the previous loving relationships, highlighting shifts in relationship roles (Carer 5).

All the time it will end up in ‘You don’t know how much I love you and I wouldn’t do anything to hurt you’… he used to always be telling me that he loved me and… I think that’s what I miss a bit really… he isn’t quite so affectionate… he used to say it on a daily basis how much he loved me and things and that was quite nice… (Carer 5)

#### Feeling used

The same four carers felt not only unloved but also ‘*used’* by their husbands for sexual gratification. This signalled to them a shift from a normal loving sexual relationship to one primarily focused on satisfying their husbands’ hypersexual needs (Carer 1).

I’ve always thought of it very old fashioned as making love… sex of sex’s sake for me is nothing… so the fact that he was then using these magazines to psych himself up to come and have sex with me was really meaning he was just using me to have sex… he was using me… like an animal really… (Carer 1)

#### Self-confidence

Three of the carers who expressed feeling unloved and used by their partners also asserted that hypersexuality and their husbands’ consequent demeanour had adversely affected their self-confidence (Carer 8).

At the time I felt completely worthless… completely and utterly worthless… I just felt so ugly and old… (Carer 8)

### Theme 11: Stigma

This theme outlines the two carer-perceived forms of stigma associated with hypersexuality: personal stigma and social stigma.

One carer’s reference to the older age group implies a stereotype that older people are less sexual, which may be used to reinforce the belief that hypersexuality is unnatural (Carer 5).

We’re in our sixties so it’s quite obvious that we’re not going to feel how we did when we first met in our thirties… but he seems to be still back in that era and wants it in the same way… (Carer 5)

Three carers expressed concerns about the social stigma associated with hypersexuality, fearing that others discovering their partner’s condition would reflect negatively on themselves and their families (Carer 1).

I suppose the thing that bothered me most was the thought that other people would find out and laugh at me because I’d always… pride always comes before a fall… I’d always been proud of my happy marriage… we’d worked at it and the thought that my husband was gay and might be discovered to be gay are… yeah… that did worry me… (Carer 1)

During interviews, carers often hesitated, laughed nervously and apologised when asked sexually specific questions or prompted to discuss their partners’ sexual experiences. This may be attributed to the embarrassment of discussing sex, concerns about crossing social boundaries and fear of being perceived as inappropriate (Carer 3).

(laughing) she’d go straight to the… not too much foreplay… not too much… normally she likes tenderness and sweetness and this was sort of a bit more lust… go for it (laughing)… (Carer 3)

### Theme 12: Professional help-seeking

This theme outlines the professional help-seeking barriers regarding hypersexuality, as well as certain aspirations with regard to professional help.

#### Barriers

Issues with seeking professional help encompassed communication barriers, lack of understanding, insufficient education, neglect by health professionals, stigma related to hypersexuality and challenges in discussing sex. All eight carers experienced difficulty obtaining adequate information and assistance for their partners’ newly developed hypersexuality, expressing frustration, sadness and anger over the unavailability of help. A key concern raised is that patients are not adequately informed about the likelihood and implications of hypersexuality when taking drugs for PD (Carer 1).

If somebody had said… well warning you that this might happen when he went on these drugs… I mean it says in the leaflets… it talks about hypersexuality… I looked at it and read the sheets through and I said ‘Oh hypersexual… he’ll be a bit frisky and that’ll be alright’… you know… the horrors of what were to come never occurred to me… if nobody speaks out then this will go on and other marriages will be ruined like mine was ruined… at least had we’ve been told it wouldn’t have been such a terrible shock… (Carer 1)

Certain carers noted a key issue: medical professionals lack knowledge about hypersexuality and show an apparent reluctance to investigate further or take patients’ and carers’ concerns seriously (Carer 5).

I have tried to broach this a few times with my husband’s neurologist… I do a bit more than hint at the problems now and again but he never sort of takes it and runs with it… we’ve been seeing him for ten years and not once has he asked about hypersexuality… or hinted… that it could be a problem… he would spend more time talking about gambling… (Carer 5)

#### Aspirations

Due to these barriers, certain carers expressed specific aspirations for professional help for individuals with hypersexuality and their carers. Over half of the carers expressed a desire for health professionals to be educated about hypersexuality and its consequences. This education is seen as a means to enable professionals to educate patients and carers about the condition, with the ultimate goals of alleviating the patient and carer burden of living with hypersexuality and facilitating more effective help-seeking behaviour (Carer 8).

(I need help) with managing the anger that I feel in a way that is useful… not in a way where somebody just sits there and tell me that my mantra should be that my husband can’t help it… I want somebody who can help me understand why I’m angry and who can help me resolve these angry feelings before my husband dies (Carer 8)

## Discussion

Using a qualitative approach, the current study aimed to explore the impact of hypersexuality on spousal carers of patients with PD and dementia. This study captured 12 themes illustrated in figure 2.

In terms of clinical phenomenology, hypersexuality manifested through changes in patients’ sexual cognitions and behaviours. These changes can be summarised using the categories presented in figure 3.

These findings resonate with existing literature on hypersexuality in neurological disorders, particularly PD and dementia. Similar sexual changes have been documented in systematic reviews, aligning with our observations.^19^ Notably, patients with PD and hypersexuality often exhibit sexual compulsivity and impulsivity,^19^,^22^ while those with dementia may show sexual disinhibition and inappropriateness.^4^ Our study partially supports this distinction, with carers of patients with FTD describing behaviours as ‘*disinhibited*,’ although overlap with sexual preoccupation and compulsivity was evident. A larger sample size might clarify these distinctions further.

Contrary to expectations, despite increased sexual urges, patients often engaged less frequently in sexual activities with partners post-onset of hypersexuality, often due to partner discontent. Patients sought gratification through masturbation, pornography, prostitution, promiscuity or affairs, influenced by partner satisfaction or absence. This association between heightened desires and actual sexual practices underscores the role of external factors, echoing literature on marital dynamics where dissatisfaction can lead to extramarital pursuits.^23^

Psychologically, carers reported disturbed moods and diminished mental health in patients, consistent with anxiety often coexisting with PD.^24^ The emotional toll on carers was profound, reflecting themes of burden and distress documented in carer literature.^25^,^27^

While all carers attributed their partners’ hypersexuality to their neurological diseases, some believed its development is linked to the patients’ past experiences. For example, Carer 1 indicated that her husband had a homosexual experience at the age of 15 with a school friend. She claimed that her husband “*might have been a repressed homosexual and the hypersexuality had overridden his control of that and was forcing him… allowing him… stimulating him to pursue the homosexuality as he had never done*” before. Carer 6 indicated that her husband had an ex-girlfriend of Indian descent during his twenties who died in a car accident. She indicated that both prostitutes her husband had been involved with, and one of whom he fell in love with, were dark-skinned and considered that there might be a link between this and the evolution of his hypersexuality. Two potential reasons for this link can be considered. First, it is possible that past behaviours had never disappeared, but rather their partners had been successful in suppressing them. These behaviours resurfaced due to neurological disorders affecting inhibitions. Second, the biological and molecular effects of medications used to manage neurological disorders, like PD, may trigger latent tendencies, although this area remains unexplored within the scope of this research.

The study revealed that hypersexuality profoundly affected carers and strained their relationships with their partners. Some carers, overwhelmed by frustration and despair in dealing with their partners’ hypersexuality, reported experiencing desires or actual instances of aggressive reactions towards their partners.

Despite efforts to cope, carers struggled with responsibility, guilt and, at times, aggressive feelings toward their partners, mirroring the challenges seen in sex addiction research.^28^,^31^ Extended discussions during assessments, with one lasting over 3.5 hours instead of the anticipated 2 hours, indicate significant distress among carers. This underscores the urgent need for support and avenues for emotional expression and sharing experiences.

The stigma surrounding hypersexuality emerged as a significant concern for carers, influencing disclosure and help-seeking behaviours. Fear of stigma led some carers to conceal hypersexuality, decline study participation or avoid healthcare appointments, reflecting broader societal discomfort with sexual topics.^32 33^ The barriers to seeking professional help include inadequate communication and education among healthcare providers, exacerbating carer distress and prolonging their silence on the issue.

### Implications

This study highlights the critical need for healthcare professionals to educate patients and carers about impulse control disorders associated with PD and dementia, including hypersexuality, and to provide ongoing support and monitoring.^4 34^ Targeted psychological and behavioural strategies could help carers manage distress and improve coping mechanisms. Acceptance and commitment therapy^35^ may be particularly beneficial, as it encourages carers to accept the challenges of their partners’ hypersexual behaviours while fostering psychological flexibility and values-based action. Group-based interventions, such as structured peer-support programmes modelled after the study by Al-Anon,^36^ could provide a shared space for carers to exchange experiences, reduce isolation and develop practical coping strategies. Additionally, cognitive-behavioural therapy tailored for carers could address maladaptive thought patterns and emotional distress related to managing hypersexual behaviours. Psychosocial interventions, including couple-based therapy and family counselling, may also facilitate communication and adaptive strategies.

### Limitations

This study encountered several limitations. First, while the sample included carers of patients with PD and FTD, the intended inclusion of carers of patients with AD was not realised. This restricted our ability to compare the impact of hypersexuality across dementia subtypes, specifically AD. Future research should prioritise recruiting a diverse sample, including carers of patients with AD, to achieve a more comprehensive understanding. Second, the study’s focus on spousal carers limited the scope of investigation. The impact of hypersexuality extends to other family members and professional carers, warranting broader investigation. Thirdly, inherent to qualitative research, response biases, such as social desirability, may have influenced participant disclosures, particularly given the sensitive nature of hypersexuality. Although a confidential and non-judgmental interview environment was established, future studies could consider incorporating anonymous surveys or mixed-methods designs to mitigate this potential bias. Finally, while this qualitative approach yielded rich, in-depth insights, a mixed-methods design, integrating quantitative analyses, would provide greater triangulation of findings and enhance the robustness of conclusions, offering a more complete understanding of the phenomenon.

### Future directions

Future research should employ mixed methods to mitigate under-reporting and explore comprehensive management strategies for hypersexuality in PD and dementia. Addressing stigma through public education and improving healthcare providers' readiness to discuss sexual health are crucial steps in supporting carers and patients alike.

### Conclusion

In conclusion, hypersexuality in neurological disorders profoundly affects patients and carers, demanding tailored interventions and support mechanisms to alleviate its emotional and psychological toll.

## Supplementary material

10.1136/bmjopen-2024-090870 online supplemental file 1

10.1136/bmjopen-2024-090870 online supplemental file 2

## Data Availability

Data are available upon reasonable request.

## References

[1] WHO (2019). International Statistical Classification of Diseases and Related Health Problems.

[2] Latella D, Maggio MG, Andaloro A (2021). Hypersexuality in neurological diseases: do we see only the tip of the iceberg?. J Integr Neurosci.

[3] Zhang J-F, Wang X-X, Feng Y (2021). Impulse Control Disorders in Parkinson’s Disease: Epidemiology, Pathogenesis and Therapeutic Strategies. Front Psychiatry.

[4] De Giorgi R, Series H (2016). Treatment of Inappropriate Sexual Behavior in Dementia. Curr Treat Options Neurol.

[5] Batla A, Tayim N, Pakzad M (2016). Treatment Options for Urogenital Dysfunction in Parkinson’s Disease. Curr Treat Options Neurol.

[6] Samuel M, Rodriguez-Oroz M, Antonini A (2015). Management of impulse control disorders in Parkinson’s disease: Controversies and future approaches. Mov Disord.

[7] Schulz R, Beach SR, Czaja SJ (2020). Family Caregiving for Older Adults. Annu Rev Psychol.

[8] Liu Z, Heffernan C, Tan J (2020). Caregiver burden: A concept analysis. *Int J Nurs Sci*.

[9] Caceres BA, Frank MO, Jun J (2016). Family caregivers of patients with frontotemporal dementia: An integrative review. Int J Nurs Stud.

[10] Soares GM, Bouça-Machado R, Abreu D (2023). Contributory Factors to Caregiver Burden in Parkinson’s Disease. Mov Disord Clin Pract.

[11] Chapman KR, Tremont G, Malloy P (2020). The Role of Sexual Disinhibition to Predict Caregiver Burden and Desire to Institutionalize Among Family Dementia Caregivers. J Geriatr Psychiatry Neurol.

[12] Tayim N, Barbosa P, Panicker J (2024). Hypersexuality in neurological disorders: A systematic review. *BMJ Ment Health*.

[13] Tayim N, Panicker JN, Foley J (2024). A qualitative study exploring the clinical phenomenology and impact of hypersexuality in patients with Parkinson’s Disease. Sci Rep.

[14] Ulin P, Robinson E, Tolley E (2005). Qualitative Methods in Public Health: A Field Guide for Applied Research. Med Sci Sports Exercise.

[15] Fusch P, Ness L (2015). Are We There Yet? Data Saturation in Qualitative Research. TQR.

[16] Guest G, Bunce A, Johnson L (2006). How Many Interviews Are Enough?:An Experiment with Data Saturation and Variability. Field methods.

[17] Braun V, Clarke V (2006). Using thematic analysis in psychology. Qual Res Psychol.

[18] Tong A, Sainsbury P, Craig J (2007). Consolidated criteria for reporting qualitative research (COREQ): a 32-item checklist for interviews and focus groups. Int J Qual Health Care.

[19] Codling D, Shaw P, David AS (2015). Hypersexuality in Parkinson’s Disease: Systematic Review and Report of 7 New Cases. Mov Disord Clin Pract.

[20] Krueger RB (2016). Diagnosis of hypersexual or compulsive sexual behavior can be made using ICD-10 and DSM-5 despite rejection of this diagnosis by the American Psychiatric Association. Addiction.

[21] Evans AH, Strafella AP, Weintraub D (2009). Impulsive and compulsive behaviors in Parkinson’s disease. Mov Disord.

[22] Isaias IU, Siri C, Cilia R (2008). The relationship between impulsivity and impulse control disorders in Parkinson’s disease. Mov Disord.

[23] Knox D, Schacht C (2016). Choices in relationships: an introduction to marriage and the family (25th edn).

[24] Chen JJ, Marsh L (2014). Anxiety in Parkinson’s disease: identification and management. Ther Adv Neurol Disord.

[25] Leroi I, Ahearn DJ, Andrews M (2011). Behavioural disorders, disability and quality of life in Parkinson’s disease. Age Ageing.

[26] Leroi I, Andrews M, McDonald K (2012). Apathy and impulse control disorders in Parkinson’s disease: a direct comparison. Parkinsonism Relat Disord.

[27] Calne SM, Lidstone SC, Kumar A (2008). Psychosocial issues in young-onset Parkinson’s disease: current research and challenges. Parkinsonism Relat Disord.

[28] Ostrowski M, Mietkiewicz MC (2015). Approach of the sexuality of Alzheimer’s disease patients according to caregivers’ guides approach. Geriatr Psychol Neuropsychiatr Vieil.

[29] Lerman SF (2012). Wellbeing of spouses of patients with Parkinson’s disease exhibiting impulse control disorders, in 19th World Congress on Parkinson’s Disease and Related Disorders, Parkinsonism and Related Disorders: Shanghai, China.

[30] Praver FC (2011). Married to a Sex Addict.

[31] Wadleigh T (2017). Sexual Addiction: Helping Spouses/Partners.

[32] Czyz EK, Horwitz AG, Eisenberg D (2013). Self-reported barriers to professional help seeking among college students at elevated risk for suicide. J Am Coll Health.

[33] Hinchliff S, Gott M, Galena E (2005). “I daresay I might find it embarrassing”: general practitioners’ perspectives on discussing sexual health issues with lesbian and gay patients. *Health Soc Care Community*.

[34] Weintraub D, Hoops S, Shea JA (2009). Validation of the questionnaire for impulsive-compulsive disorders in Parkinson’s disease. Mov Disord.

[35] Hayes SC, Luoma JB, Bond FW (2006). Acceptance and commitment therapy: model, processes and outcomes. Behav Res Ther.

[36] Kverme A (1990). Al-Anon. A resource for families and friends of alcoholics. Tidsskr Nor Laegeforen.

