# Supplementary material for: Impact of hypersexuality on spousal carers of patients with Parkinson’s disease and frontotemporal dementia: a qualitative study
Source: BMJ Open. 2025 Apr 10;15(4):e090870. doi: 10.1136/bmjopen-2024-090870 (PMC11987135; doi:10.1136/bmjopen-2024-090870)
Supplement: online supplemental file 1 [file bmjopen-15-4-s001.docx]

**Carer Assessment Interview
Semi-structured interview schedule**

*Please note that not all carers are necessarily partners; therefore, there are some interview questions that can only apply to partners. Questions that only apply to partners are under a separate heading.*

Interview length: 35-60 minutes
*About the patient (*to be extracted from patient notes)

Age of patient:
Neurological disorder of the patient:
Age of onset of neurological disorder:

Date:
Time:

**INTRODUCTION**
Thank you for agreeing to take part in an interview for this project.

This interview will be audio recorded. The main reason for this is to have an accurate set of data on this topic. This will help researchers analyze the data as the project develops. Rest assured that you would remain completely anonymous. All data collected is confidential. No records of the interview will be kept with your name or the name of the patient on it.

The following sections include questions about increased sexual behavior that has happened since the patient has developed (insert name of neurological disorder). This is called hypersexuality. Please remember that sexual acts involving physical harm to others or child abuse is against the law. For this reason, please do not answer any questions that show that the patient’s sexual behavior has been a threat to others or that the patient has had sexual relationships with minors.

I understand how sensitive this topic is. If any questions make you uncomfortable, you are completely free not to answer, but we would be grateful if you can answer all questions. Also, if any questions are not understandable, please ask and they will be explained.

**GENERAL BACKGROUND**

1. **Question:** How old was the patient when they first became hypersexual?
2. **Question:** What is your relationship to the patient?
   **Probe 1:** How long have you been in this relationship?
   **Probe 2:** (if applicable) When did the relationship end?

**Probe 3:** Was the hypersexuality a reason for the end of your relationship?

1. **Question**: Did the patient have any behavioral or cognitive disorders before the (insert name of neurological condition)?
   Example of behavioral disorder is obsessive-compulsive disorder.
   Example of cognitive disorder is perception and memory disorders.
   **Probe:** Can you tell me what they are?
2. **Question:** Does the patient have any previous addictions, such as drugs or alcohol?
   **Probe:** What addictions?
3. **Question:** Did/does the patient have any other impulse control disorders such as increased gambling behavior, increased eating behavior, or increased buying behavior?
   **Probe 1:** Which ones?
   **Probe 2:** When did they start?
   **Probe 3:** How severe were/are these behaviors?
4. **Question:** Did/do you notice any other changes in the patient’s behavior apart from these and the hypersexuality?
   **Probe 1**: What are they?
   **Probe 2:** When did these changes start?
5. **Question:** Did you notice that the hypersexuality developed after use of any medications?
   **Probe:** What medications?

**SPECIFIC**

1. **Question:** When did you first notice this increased sexual behavior?
   **Probe 1:** When you first noticed this behavior, how did you feel?
   **Probe 2:** Is the patient still showing this behavior?
2. **Question:** Do you believe the patient developed hypersexuality because of (insert name of neurological disorder)?
   **Probe:** Why do you think so?
3. **Question:** Since the patient’s (insert name of neurological disorder) started, did/do you feel the patient has lost interest in sex in general?
   **Probe:** What makes you think so?
4. **Question:** Since the hypersexuality started, do you believe the patient has new sexual interests that were not there before the (insert name of neurological disorder)?
   **Probe 1:** What are the new interests?
   **Probe 2:** How did you notice them?
5. **Question**: How much time do you think the patient spent/spends on their new sexual interests?
6. **Question:** Since the hypersexuality started, do you believe that your physical relationship with the patient has changed?
   **Probe:** Can you tell me how?
7. **Question:** Since the hypersexuality started, has the patient become more interested in sex with you?
   **Probe:** What is your reaction?
8. **Question:** Since the hypersexual behavior started, do you think the patient had/has no control over their hypersexuality?
   **Probe:** What makes you think so?
9. **Question:** Since the hypersexual behavior started, do you feel like the only thing the patient could/can think about is sex?
   **Probe:** What makes you think so?
10. **Question:** Does the patient’s hypersexuality cause problems in your relationship?
    **Probe 1:** Can you please give elaborate? What kind of problems?
    **Probe 2:** How does this make you feel?
    **Probe 3:** How do you think this makes the patient feel?
11. **Question:** Do you believe the patient was/is more tempted to engage in sexual behavior when they have certain feelings, such as sadness or anxiety?
    **Probe:** What feelings?
12. **Question:** Which of the following has your partner tried since developing hypersexuality? I will list them and you are required to just say yes or no to each.
    Internet porn?
    Pornographic novels?
    Uncontrollable masturbation?
    Prostitution?
    Voyeurism: getting sexual satisfaction from spying on sexual objects or acts?
    Exhibitionism: the act of showing your genitals to strangers?
    Affairs?
    Anonymous sexual encounters?
    One-night stands?
    Bath houses: communal bath places?
    Massage parlors?
    Strip clubs?
    Sexual encounters with gender not typically interested in?
    Sexual misconduct in the workplace?
    Being aggressive with sexual partner?
    Asking for sexual partner to be aggressive?
    Bestiality: sexual encounters with animals?
    Any others that I haven’t listed?
13. **Question:** Do you think the hypersexuality has negatively affected the patient’s life?
    **Probe:** Has it affected their

Marital life? How so?

Family life? How so?

Social life? How so?

Work? How so?

Finances? How so?

Health? How so?

Mood? How so?

Sleep? How so?

Self-confidence? How so?
Quality of life? How so?

1. **Question:** To your knowledge, has the patient tried to control their sexual behavior or stop it altogether?
   **Probe 1:** Has it been successful?
   **Probe 2:** How does this make the patient feel?
2. **Question:** To your knowledge, does the patient want to overcome their hypersexuality?
   **Probe:** How can you tell?
3. **Question:** Did the patient ever seek advice for their sexual behavior?
   **Probe:** What was the result of that?
4. **Question:** How did/does the patient’s hypersexuality make you feel?
   **Probe 1:** Do you think the patient knows this?
   **Probe 2:** Have you tried to make them aware?
   **Probe 3:** What has been the patient’s reaction?
5. **Question:** Do you believe the hypersexual behavior was/is out of the patient’s control?
   **Probe 1:** Did/do you discuss this issue with the patient?
   **Probe 2:** What has resulted from those conversations?

**PARTNER QUESTIONS**

1. **Question:** Since the hypersexual behavior started, did/do you feel there was/is less intimacy and confidence between you and your partner when you have sex?
   **Probe:** Why do you think this has happened?
2. **Question:** Since the hypersexual behavior started, did/do you feel your partner was/is not sexually interested in you anymore?
   **Probe 1:** How does this make you feel?
   **Probe 2:** Have you talked to your partner about this?
   **Probe 3:** What did they reply?
3. **Question:** Before the patient’s (insert name of neurological condition) started, how often did you and your partner have sex?
4. **Question:** In the period between the start of the patient’s (insert name of neurological condition) but before the start of hypersexuality, how often did you and your partner have sex?
5. **Question:** Since the hypersexuality started, how often do you and your partner have sex?

1. **Question**: Did/do you find your partner repulsive?
2. **Question**: Did/do you feel you lost respect for him?
3. **Question**: Do you think you will ever be able to forgive him?
4. **Question:** Do you ever blame yourself for the patient’s hypersexuality?

**CLOSURE**We have reached the end of our interview. I would like to thank you for being so patient. However, do you believe there is anything we have missed out that you would like to add?
Do you have any other comments about what we have discussed, or about the research as a whole?
We will send you a summary of the research findings when it becomes available.
Thank you so much for your participation.
