# Supplementary material for: Impact of hypersexuality on spousal carers of patients with Parkinson’s disease and frontotemporal dementia: a qualitative study
Source: BMJ Open. 2025 Apr 10;15(4):e090870. doi: 10.1136/bmjopen-2024-090870 (PMC11987135; doi:10.1136/bmjopen-2024-090870)
Supplement: online supplemental file 2 [file bmjopen-15-4-s002.docx]

**Table S1.** Emergent themes, subthemes, and quotes analyzed for Parkinson’s disease/frontotemporal dementia carers.

| **Themes** | **Subthemes** | **Quotes** |
| --- | --- | --- |
| **Theme 1: Manifestations** | **1.1. Indicators** | “I found a till receipt for a gay magazine… I sat on the knowledge for a couple of weeks but first of all I went straight up to WH Smith and bought a copy of the magazine thinking that either it wasn’t what I thought it was… it was Gay Times… or this had been bought by mistake… I got a copy… I sat there outside and read it and realised it was highly unlikely that it had been bought by mistake…” (Carer 1) |
|  | **1.2. Desires** | “That’s the only thing he’s interested in … to have sex…” (Carer 7) |
|  |  | “I don’t know where desire’s gone because it is practically... it is non-existent… they happened about the same time I think... I think it must be... it must be a good five or six years as far as he’s concerned... and I honestly think that things changed for me around sixty...” (Carer 5) |
|  | **1.3. Behaviors** | “Normally she likes tenderness and sweetness and this was sort of a bit more lust… go for it… behavior was extreme if you like because she’s a reserved person … who has other high standards of good behavior… so this was like nature in the raw really…” (Carer 4). |
|  | **1.4. Preoccupation** | “her thoughts are uncontrollable and come so much of the time…” (Carer 3) |
|  | **1.5. Compulsivity** | “Hypersexuality is present all throughout the day and during the night while I am asleep…” (Carer 3) |
| **Theme 2: Sexual practices** | **2.1. Practices with the partner** | “And now [he was asking for sex] every morning… every evening… sometimes he’s asking during the day…” (Carer 7) |
|  |  | “She didn’t ask for Fifty Shades of Grey no… but still … a little hint of S&M which really wasn’t part of our repertoire…” (Carer 4) |
|  |  | “Things like going outside the door and knocking on the door and coming in or something… you know… I’m somebody he’s picked up outside or something and who knocks on his door and slips in with exotic underwear on or something… never had all this before… it’s just weird… like he was sort of switched off… he’s actually thinking he’s with a prostitute or something I don’t know…” (Carer 5) |
|  | **2.2. Practices with themselves** |  |
|  | **2.3. Practices with others** |  |
|  | **2.4. Deviant sexual behavior** | “It needs to be more upfront that it’s not just about a decrease in sex or an increase in sex… it could be a decrease in a normal sexual relationship and a… a subverted or a hidden cover increase in some kind of deviant sexual behavior which had been what was going on for twenty years and I didn’t know about…” (Carer 8) |
| **Theme 3: Impact** | **3.1. Marital life** | “It was dreadful… devastating … I couldn’t make head and the tail of it… it just didn’t add up to the man I’d been living with for nearly fifty years…” (Carer 1) |
|  |  | “It’s awful really because he’s not the same person… apart from everything else that’s going on… I feel like I’m sort of living a double life and I sort of have to live his life as well and double check everything … life’s so difficult so it’s not surprising that I’m tired…” (Carer 5) |
|  |  | “I’ve always thought of it very old fashioned as making love… sex for sex’s sake for me is nothing… so the fact that he was then using these magazines to psych himself up to come and have sex with me was really meaning he was just using me to have sex...” (Carer 1) |
|  |  | “It’s kind of became more of ritual… more of a… I mean he would say things like ‘I need a fuck’… like every morning and every evening… I have felt really pursued… that’s the only thing he’s interested in… is to have sex…” (Carer 7) |
|  |  | “It’s difficult to separate if it’s the dementia or more the sexual aspect of it… it’s kind of loss of companionship in all areas so it has affected the relationship…” (Carer 7) |
|  |  | “I’m competing with the women on television… sometimes I’m thinking… does he think that he’s making love to me or does he think he’s making love to somebody off the television…” (Carer 5) |
|  |  | “I’ve lost respect for him… how can you respect someone that gets off of watching little boys being humiliated… I’ve said to him I won’t collude or condone with anything he’s done… and I won’t accept those things either… and that whilst he lives in the house with me he behaves in a way I would want him to behave legally...” (Carer 8) |
|  | **3.2. Family, social life and daily activities** | “My kids were shocked, so mentally and emotionally distanced themselves…” (Carer 1) |
|  |  | “The children just could not understand it… he never denied it… both the children were irritable… they couldn’t understand it you know because [of] the way he’d been brought up and how he’d brought them up…” (Carer 6) |
|  |  | “I decided that if he agreed… and he did agree… that I would take his credit and debit cards off him… hide any money I’d got in the house… I left him… I think we agreed on fifteen pounds which would be enough for taxi and whatever so he couldn’t do that… I hid the cheque books and hid any money I’d got in the house so he didn’t have any access to cash… and that worked well for a couple of months and then he remembered that he had an account that I’d forgotten about…” (Carer 1) |
|  | **3.3. Health and well-being** | “He couldn’t sleep because I was working so hard and he was messing around… he said he cried most nights…” (Carer 6) |
|  |  | “He was anxious and depressed… worried about everything…” (Carer 1) |
|  |  | “He was more stressed because he just couldn’t understand what he was doing…” (Carer 6) |
|  |  | “He seemed very withdrawn… he was completely locked into this mad behavior… there was no happiness… there was no joy… he never smiled… he was apathetic… he was almost irritable… he was quite angry… he doesn’t get irritable… he doesn’t show it… if he is and this is what frightens me about him… I feel it’s like watching a pressure cooker and there’s going to be a time when it pops…” (Carer 8) |
|  |  | “Probably more confident… I mean she was writing at the time… that’s her identity… she’s a writer…” (Carer 4) |
|  |  | “When I asked him when he stopped loving me … he said he didn’t know and he eventually said ‘I think I’m narcissistic and I’m in love with myself…” (Carer 8) |
|  |  | “I think that as with the madness of love or something… it raises you up but it also is a madness so it is a sickness… it is a sickness we all enjoy…” (Carer 4) |
|  |  | “I knew he was dating again… he’d go out looking really quite handsome in something that I’d suggested to upgrade his wardrobe… go out looking attractive…” (Carer 6) |
| **Theme 4: Control** | **4.1. Loss of control** | “He couldn’t resist it… it was hopeless… he couldn’t stop it…” (Carer 1) |
|  |  | “It’s become like a bit of a habit… like something he asks for… it’s a bit like asking for a bit more wine…” (Carer 7) |
|  |  | “There is a difference… the impulse to do something and the ability to know right from wrong… he knows what’s right and what’s wrong but he chose to take a risk and his risk-taking has increased… he is the one with his hand on his penis…” (Carer 8) |
|  |  | “I think she probably hadn’t got [control]… I think she probably felt a bit out of control… but she didn’t seem distressed…” (Carer 4) |
|  | **4.2. Attempt to reduce/stop** | “I think he’s doing a good job in trying to keep a lid on it… it’s still there but more controlled…” (Carer 2) |
|  | **4.3. Desire to overcome** | “[He] desperately wanted to stop it… he just couldn’t work out what had hit him…” (Carer 1) |
|  |  | “[It] absolutely drives her mad and does not make her happy… if clitoris removal existed she would have gone for it…” (Carer 3) |
|  |  | “I don’t think that he admits that he’s hypersexual… because whenever it’s come up like now or even when the neuropsychologist was there… it’s not something that he’d actually readily say ‘Yes I have got a problem’… I don’t think he thinks he’s got a problem…” (Carer 5) |
|  |  | “I don’t think he understands actually…” (Carer 7) |
| **Theme 5: Emotional formulations** | **5.1. Around hypersexuality** | “I just didn’t know what had happened ... it’s like waking up on the other side of the mirror like Alice in... Through the Looking Glass... it was just so abnormal... he was cold towards me…” (Carer 8) |
|  |  | “I was shocked... I couldn’t make head and the tail of it... it just didn’t add up to the man I’d been living with for nearly fifty years…” (Carer 1) |
|  |  | “Normally she likes tenderness and sweetness and this was sort of a bit more lust... go for it... [laughing]... and in a way that was fresh and amusing... again one took that as a positive thing... for a while anyway…” (Carer 4) |
|  | **5.2. Around partner** | “I just felt really sorry for him… the only pleasure he has in life is to have sex so I didn’t find it difficult for me to… you know… have sex with him… because I felt sorry for him… it was fine for me as well…” (Carer 7) |
|  |  | “I was so angry… it wasn’t just emotion… there was anger… I felt very angry about what he’d done … I wouldn’t want him to touch me because I don’t know who he is… he was doing things that are completely unacceptable… sad… I was very sad… I felt rejected… I felt confused… I feel such a fool… let down…” (Carer 8) |
|  |  | “I was a bit unquestioned maybe looking back… it wasn’t that extreme you know… it was extreme if you like because she’s a reserved person who you know… who has other high standards of good behavior you know… so this was like nature in the raw really… which didn’t in the least turn me off…” (Carer 4) |
| **Theme 6: Beliefs in the causes of hypersexuality and attributions** | **6.1. Self-blame** | “The longer he’s not having sex the worse it’s making him… so basically that might be my fault…” (Carer 5) |
|  | **6.2. Blame on neurological disease and/or its management** | “I suppose now I can point to Ropinirole and say it’s Ropinirole’s fault…” (Carer 5) |
|  |  | “He was already on this medication then so you know... and we tried to work out which it was... I thought it was when the entacapone had been added…” (Carer 4) |
|  |  | “Part of the pain in the neck of the disease… awfulness of package that’s changed our lives…” (Carer 3) |
|  |  | “I think it just came with the disease… right before he passed I said to him ‘You couldn’t help it… it wasn’t you… it wasn’t what you were like… it was a disease and you’ve got two of them and they’re both serious’…” (Carer 6) |
|  |  | “I recognised that it isn’t his fault… it doesn’t make it any easier to bare…” (Carer 8) |
|  | **6.3 Blame on partners and their past experiences** | “[Husband’s] parents were away… he was allowed… for a night… and he was allowed to ask his friend from his school to stay overnight which he did… and then some sort of homosexual activity occurred… I mean the implication has always been that he was a repressed homosexual and the hypersexuality had overridden his control of that and was forcing him... allowing him... whatever... stimulating him to pursue the homosexuality as he never had done as far as I know...” (Carer 1) |
| **Theme 7: Relationship with the partner** | **7.1. Impact on marriage** | “It’s not like an intimate loving relationship… it’s more mechanical and ritual-like…” (Carer 7) |
|  |  | “Hypersexuality is his way of being masculine… not for sexual gratification but rather for me to enjoy it as well… but he doesn’t understand that I don’t…” (Carer 2) |
|  |  | “It’s not making love to me or me making love to him in the way that I used to know… it’s not that anymore…” (Carer 5) |
|  |  | “I stopped being a wife and became a housekeeper and a carer…” (Carer 1) |
|  |  | “I’m just there to put food on the table… to clean the house… and he’s polite to me because that’s how he’s been brought up… to be polite… but it’s not a marriage…” (Carer 8) |
|  |  | “I actually feel now that I’m… it’s a role reversal... I don’t think he’s looking after me… I think I’m looking after him…” (Carer 5) |
|  |  | “I half felt amused in a way because I don’t really feel insecure … you know… it’s a good relationship…” (Carer 4) |
|  | **7.2. Image of partner** | “It just didn’t add up to the man I’d been living with for nearly fifty years…” (Carer 1) |
|  |  | “I’m losing the husband that I had… he’s just not the same anymore…” (Carer 5) |
|  |  | “The man I married was intelligent… vibrant… really really fun to be with… very very loving… I’m now living with not just the fact that I lost my husband but that my husband was never who I thought he was… I don’t know who this person is and in fact I got him to move out of our bedroom the night I found out about the pornography… and I lay in bed that night on my own… he was in the other room… and I had the duvet and my arms underneath and I thought ‘Put your arms on top’ and then I thought ‘Why did I think that?’ and I thought ‘Because he might come in… I’m frightened’ then I got up and I locked my bedroom door… because I was so frightened of who this person was because he was not the man I married and I now had proof he was not the man I married… this is a man who was having to imagine he was wearing women’s clothes before he could get an erection with me… who is this man and did I ever know? It made me question everything…” (Carer 8) |
|  | **7.3. Aggression** | “I think the worst thing was that on one occasion I actually momentarily considered violence towards him… he’d had one of his trips to the sex shop… he got stuff… I’d been out in the garden… and I’d seen him through the window of his office… obviously he was busy looking at some stuff… and it was lunch time and I came in to give him his lunch… and I stood behind him and I really can’t believe it now but I’ve got to tell you… it’s the truth… I stood behind him with this big hammer in my hand… and I thought quite clearly ‘A couple of blows to your skull with this and this would all be over’… and then I put the hammer away and served his lunch…” (Carer 2) |
|  |  | “Even after I’d found out, I couldn’t get him to talk to me about it and I remember going to his workshop one day like this and I asked him and asked him and he just stood there like a defiant little boy… and I picked him up… I’m only five feet… he’s five foot six… he’s much bigger… I picked him up by his boiler suit and I walked him backwards to the wall… just lifted him off the floor… banged him against the wall and I said ‘Talk to me’ and he just stood there till I let go of him… nothing moves him… nothing moves him… my GP said ‘Make sure you’re not near the knife block when you do hit him… get out of the kitchen’… she said ‘Don’t put yourself in danger’ and what she meant was danger of being arrested I think…” (Carer 8) |
| **Theme 8: Dealing with hypersexuality** | **8.1. Attempt to limit hypersexuality** | “If he did continue to do something and the police came… I would step aside… I would explain that he had a degenerative brain disease but I’m not going to protect him if he’s doing something illegal which he was… I think there’s a limit to how much protection I can afford someone who has done nothing to deserve protection…” (Carer 8) |
|  |  | “I would switch the television off and take the [pornographic] DVD out… I think I became very controlling... and I’m not sure if that was the right thing or the wrong thing to do but I did… for a start because I found it offensive… very offensive…” (Carer 1) |
|  |  | “I don’t like going to bed first because I lay in bed trying to listen whether he’s changing channels… whether it’s really Match of the Day or something else… and he started going to the second living room a bit too… the guest accommodation next door… he goes in there occasionally and says it’s because he wants to watch something different to what I’m watching and then he starts putting the DVD player on… and again it’s probably all okay but I think maybe… has he got some funny DVD or something…” (Carer 5) |
|  | **8.2. Attempt to uncover facts about hypersexuality** | “I certainly looked for materials he’d obtained and was using… when I realised that he had bought a gay magazine… because I found the receipt by chance… after it when I thought about it he just said he was just curious… when he was out I went and unlocked the case and found more magazines… so yes I did go looking for them… yes I did go and look in his case and see what he’d got…” (Carer 1) |
|  |  | “Partly I snooped... when I saw two thousand pounds being taken out of... you don’t just take that out... but partly I did a ring back... a 1471 and got connected to the sex line on our phone... I mean he didn’t bother to disguise it because I don’t think he could…” (Carer 6) |
|  |  | “He goes into day care two days a week… I search the room… I look under the mattress… I look under the carpet… I look inside the pillowcases… it’s turned me on to being hyper vigilant…” (Carer 8) |
|  | **8.3. Giving in to hypersexuality** | “I thought ‘God this poor man has been a repressed gay all his life… he’s never indulged in it… I know he’s ill… he hasn’t got that many more years to live… if he wants to indulge in this why shouldn’t he?’ and so I said to him ‘Look you can’t drive now… if you want to go to gay bars and clubs I will take you there’… after you’d phoned me and said that there is some evidence that it does alter sexual orientation… I just sat and cried… I thought ‘Poor man’… he must’ve been so confused with what’s happening to him… utterly… and he couldn’t resist it…” (Carer 1) |
|  |  | “Not like I feel it’s a great suffering to me… it’s… to me… about his needs… maybe more than mine…” (Carer 7) |
| **Theme 9: Coping with hypersexuality** | **9.1. Responsibility/guilt** | “I thought I had done something and I tried for twenty years to find out what it was and when I found out it had all been him I didn’t feel responsible…” (Carer 8) |
|  |  | “I sort of think well [laughing] maybe it is my fault... maybe it is my fault that you know I’m not... wanting to have sex every night or whatever... I don’t know where desire’s gone because it is practically... it is non-existent…” (Carer 5) |
|  | **9.2. Understanding the hypersexuality** | “Kind of owning the fact that… that sex is not just with the other… it’s your relationship with yourself as well as the other person so I’m able to separate how to be who I am and who he is so I don’t actually feel exploited… like I’m able just to see that he has a greater need for sex than me and for our relationship to work I help him to meet that need and I’m having lots of other needs met in our relationship… it balances quite nicely…” (Carer 7) |
|  |  | “I think I put it down to her transference and the peculiar relationship that is actually truly expected within... within a serious therapeutic relationship... I mean it is a relationship of huge power... and... I think in a way she was supposed to have this transference... I think that was part of the deal... he was meant to become her father and she felt a sort of way towards her father…” (Carer 4) |
|  |  | “After you’d phoned me and said that there is some evidence that it does alter sexual orientation... I just sat and cried... I thought ‘Poor man’... he must’ve been so confused with what’s happening to him... utterly... and he couldn’t resist it…” (Carer 1) |
|  |  | “She [GP] just let me cry and she said to me ‘You know… you’re always going to feel sad about this’… she didn’t try and pretend it would go away… I said to her ‘That’s the most genuine response I’ve had so far’…” (Carer 8) |
|  | **9.3. Forgiveness** | “on the road to forgiveness” (Carer 1) |
|  |  | “some things can’t be unsaid” (Carer 8) |
|  | **9.4. Difficulties with coping** | “[I am] further back than I have ever been because I don’t feel that safety and security that I feel I need to have” (Carer 2) |
|  |  | “I just wished I didn’t exist” (Carer 2) |
|  |  | “[I] didn’t want to commit suicide but I would like not to exist and there’s a difference between not wanting to exist and wanting to be dead…” (Carer 8) |
| **Theme 10: Self-image** | **10.1. Feeling unloved** | “I feel as if he is only interested in me sexually…” (Carer 2) |
|  |  | “All the time it will end up in ‘You don’t know how much I love you and I wouldn’t do anything to hurt you’… he used to always be telling me that he loved me and… I think that’s what I miss a bit really… he isn’t quite so affectionate… he used to say it on a daily basis how much he loved me and things and that was quite nice...” (Carer 5) |
|  | **10.2. Feeling used** | “I’ve always thought of it very old fashioned as making love… sex of sex’s sake for me is nothing… so the fact that he was then using these magazines to psych himself up to come and have sex with me was really meaning he was just using me to have sex… he was using me… like an animal really…” (Carer 1) |
|  |  | “I feel… I’m competing with the women on the television or in his mind… I feel like he wants me to be one of them rather than… being me…” (Carer 5) |
|  |  | “He has said he had had to imagine he was wearing women’s clothes before he could get an erection with me and that makes me feel really creepy because I was in bed with someone who was going to imagine he was wearing women’s clothes before he could touch me…” (Carer 8) |
|  | **10.3. Self-confidence** | “On one occasion I said to my husband ‘I don’t understand how you can do this to me’… I’ve always stayed slim… I was always reasonably dressed… I was his official wife… had to go to functions and things with him… he always said how well dressed I looked… I could talk to people and do the proper job as a wife… that he had never been short of sex… so what was it?” (Carer 1) |
|  |  | “At the time I felt completely worthless… completely and utterly worthless… I just felt so ugly and old…” (Carer 8) |
|  |  | “[My] counselling training has helped me to be more confident in who I am so it doesn’t rattle me as much as it might other people…” (Carer 7) |
| **Theme 11: Stigma** | | “We’re in our sixties so it’s quite obvious that we’re not going to feel how we did when we first met in our thirties… but he seems to be still back in that era and wants it in the same way…” (Carer 5) |
|  |  | “I suppose the thing that bothered me most was the thought that other people would find out and laugh at me because I’d always... pride always comes before a fall... I’d always been proud of my happy marriage... we’d worked at it and the thought that my husband was gay and might be discovered to be gay are... yeah... that did worry me...” (Carer 1) |
|  |  | “I can’t really spread the word because... I would... but because of the children and the embarrassment of you know having a father do that which is difficult …. Someone such as myself who has been through it... I’m actually quite free to talk about it away from home and I’m quite happy to talk about it away from home…” (Carer 6) |
|  |  | “[laughing] she’d go straight to the… not too much foreplay… not too much… normally she likes tenderness and sweetness and this was sort of a bit more lust… go for it [laughing]…” (Carer 3) |
|  |  | “I mean the change was there in just the amount of sex we were having and the sort of… you know… on the stairs as it were you know… which wasn’t something we’d done for many years not since our young days… sorry…” (Carer 3) |
|  |  | “I’m being horribly honest here… is this alright?” (Carer 1) |
| **Theme 12: Professional help-seeking** | **12.1. Barriers** | “If somebody had said… well warning you that this might happen when he went on these drugs… I mean it says in the leaflets… it talks about hypersexuality… I looked at it and read the sheets through and I said ‘Oh hypersexual… he’ll be a bit frisky and that’ll be alright’… you know… the horrors of what were to come never occurred to me… if nobody speaks out then this will go on and other marriages will be ruined like mine was ruined… at least had we’ve been told it wouldn’t have been such a terrible shock…” (Carer 1) |
|  |  | “I have tried to broach this a few times with my husband’s neurologist… I do a bit more than hint at the problems now and again but he never sort of takes it and runs with it… we’ve been seeing him for ten years and not once has he asked about hypersexuality… or hinted… that it could be a problem… he would spend more time talking about gambling…” (Carer 5) |
|  |  | “No one cares enough … you just don’t feel listened to … the overwhelming feeling is of not being believed … even neurologist, even psychoneurologists… don’t know enough about it” (Carer 6) |
|  | **12.2. Aspirations** | “necessity of full disclosure” (Carer 4) |
|  |  | “[hypersexuality] has to become a specialty… I wish that they wouldn’t say to go to marriage guidance and counselling because… they are not equipped to handle [it]” (Carer 6) |
|  |  | “[I need help] with managing the anger that I feel in a way that is useful… not in a way where somebody just sits there and tell me that my mantra should be that my husband can’t help it… I want somebody who can help me understand why I’m angry and who can help me resolve these angry feelings before my husband dies” (Carer 8) |
