# Supplementary material for: Impact of hypersexuality on spousal carers of patients with Parkinson’s disease and frontotemporal dementia: a qualitative study
Source: BMJ Open. 2025 Apr 10;15(4):e090870. doi: 10.1136/bmjopen-2024-090870 (PMC11987135; doi:10.1136/bmjopen-2024-090870)
Supplement: Supplementary file 3 [file bmjopen-2024-090870.reviewer_comments.pdf]

PEER REVIEW HISTORY

BMJ Open publishes all reviews undertaken for accepted manuscripts. Reviewers are asked to complete a checklist review form and are provided with free text boxes to elaborate on their assessment. These free text comments are reproduced below.

ARTICLE DETAILS

Title (Provisional)

The Impact of Hypersexuality on Spousal Carers of Patients with Parkinson’s Disease and Frontotemporal Dementia: A Qualitative Study

Authors

Tayim, Natalie; Panicker, Jalesh; Foley, Jennifer; Selai, Caroline; El Sheikh, Walaa

---

VERSION 1 - REVIEW

**Reviewer**	**1**
**Name**	**Pedroso, Janari**
**Affiliation**	**Universidade Federal do Pará, Programa de Pós-Graduação em Teoria e Pesquisa do Comportamento**
**Date**	**26-Jul-2024**
**COI**	**No**

The article presents a significant contribution to understanding the impact of hypersexuality on caregivers of patients with neurodegenerative diseases. However, several aspects could be improved to strengthen the validity and applicability of the findings.

Limited Sampling and Diversity

One of the main weaknesses of the study is the small sample size, comprising only eight caregivers, with five caring for patients with Parkinson's Disease (PD) and three with Frontotemporal Dementia (FTD). This small sample size limits the generalisability of the results, restricting the representativeness of the caregivers' experiences. It would be important to use a criterion of content saturation to define the sample size (Guest G, Namey E, Chen M (2020) A simple method to assess and report thematic saturation in qualitative research. PLoS ONE 15(5): e0232076. https://doi.org/10.1371/journal.pone.0232076).

Data Collection Method

The semi-structured interviews, lasting from two to four hours, may have caused participant fatigue, potentially affecting the quality and depth of responses. Long interviews can be exhausting, resulting in less detailed or consistent responses. How did the authors acknowledge and address this issue?

Detailing of Thematic Analysis

While thematic analysis is an excellent approach, the article could have provided a more detailed description of the coding and categorisation process of the qualitative data. The lack of details on the analysis process may raise questions about the study's transparency and replicability. Including a more comprehensive description of the coding process, with examples of interview excerpts illustrating each theme, would enhance the transparency and reliability of the results.

Discussion of Limitations

The study's limitations section is adequately addressed but could be expanded to discuss methodological limitations and their implications more deeply. A more detailed discussion would help better understand the potential biases and limitations of the study, such as the influence of response biases and the limitation in generalising the results.

In conclusion, while the article offers valuable insights into the impact of hypersexuality on caregivers, methodological improvements, sample expansion, and greater detail in the analysis and discussion would significantly strengthen the research. These enhancements would increase the practical applicability of the findings, contributing to better support for caregivers and patients affected by hypersexuality.

**Reviewer**	**2**
**Name**	**Duits, Annelien**
**Affiliation**	**Maastricht University Medical Centre+ Brain+Nerve Center**
**Date**	**09-Feb-2025**
**COI**	**no**

Hyperseksuality is a relevant topic especially with respect to caregiver burden. However, in my opinion, the overall impact of hypersexuality is already well-known, perhaps not at the level of the individual caregiver but certainly at that of family caregivers in general (e.g., stigma, frustration) and the quality of the relationship with the person with Parkinson's disease (PD) and dementia. The type of hypersexuality such as the more impulsive and compulsive type in PD versus disinhibition and inappropriateness in dementia (FTD in particular) has more news value and needs more attention in the discussion. In addition to a larger sample size or other methodological issues, the underlying mechanisms could also be addressed here.

Although there is little new information, the paper is well written and qualitative studies are very welcome to outline the impact of a problem.

Some suggestions for improvement of the present paper are:

Please explain why it was not possible to involve patients in designing the study.

Refer to the supplemental info about the carer assessment interview, now a reference in the text is lacking, while the interview has been added to the manuscript.

Please ad a paragraph on rigour and reflexivity, see the Consolidated Criteria for Reporting Qualitative Research (COREQ) (Tong, Sainsbury, and Craig, 2007)

Consider figure 2 and 3 as part of the results instead of the discussion.

Discuss the small sample size with respect to the criteria of qualitative research (individual interviews) such as saturation, homogeneity of the sample etc. The present sample is relatively heterogenous given the different diagnoses. No information has been given about saturation and besides that saturation is a debatable issue. See for instance Vasileiou, K., Barnett, J., Thorpe, S. et al. 2018)

Finally discuss the added value (unless limitations) of the present findings. Are the findings helpful in designing treatments other than education?

VERSION 1 - AUTHOR RESPONSE

Reviewer: 1
Dr. Janari Pedroso, Universidade Federal do Pará
Comments to the Author:
The article presents a significant contribution to understanding the impact of hypersexuality on caregivers of patients with neurodegenerative diseases. However, several aspects could be improved to strengthen the validity and applicability of the findings.
Limited Sampling and Diversity
One of the main weaknesses of the study is the small sample size, comprising only eight caregivers, with five caring for patients with Parkinson's Disease (PD) and three with Frontotemporal Dementia (FTD). This small sample size limits the generalisability of the results, restricting the representativeness of the caregivers' experiences. It would be important to use a criterion of content saturation to define the sample size (Guest G, Namey E, Chen M (2020) A simple method to assess and report thematic saturation in qualitative research. PLoS ONE 15(5): e0232076. https://doi.org/10.1371/journal.pone.0232076).

Response:
Thank you for your comment. One of the most debated issues in qualitative interviewing and analysis is whether it is possible, or even desirable, to estimate the number of interviews to conduct prior to undertaking them and the number of interviews required to reach "saturation". Saturation can be defined as the point researchers reach whereby more data would no longer be of benefit in answering the research questions (1, 2). A study by Lee et al. (2002) suggests that studies using more than one method requires fewer participants (3). Further, a paper by Baker and Edwards (2012) published by the National Centre for Research Methods reports a study that included 14 renowned social scientists and 5 early career researchers who were all asked the question "How many qualitative interviews is enough?"

(4). The consensus was generally that “it depends on the purpose of the research” (4). The below table reports some of the answers:

“How many qualitative interviews is enough”: Responses from paper by Baker and Edwards (2012)

Expert	Response
Adler and Adler (p. 8)	12-60 interviews (mean = 30)<br>Conducting too many interviews may not only be unnecessary and time consuming, but may also reduce the time given to the analysis of data consequently reducing the quality of the analysis
Becker (p. 15)	One interview may be enough to give a rich account of a unique event<br>A few may be enough if the purpose is to highlight that an issue is more complex than previously though
Bryman (p. 18)	>20 interviews for purely interview-based studies
Charmaz (p. 21) and Doucet (p. 25)	Depends on career stage of researcher, their analytical ambitions, and the community reviewing the study
Jenson (p. 39)	Depth and complexity of analysis matter more than sample size
Mason (p. 29)	Cautions against “knee-jerk reaction” that more interviews are always better

When compared to quantitative studies, yes - the sample size presented is small (relatively); however, for qualitative research, it is sufficient enough to generate theory and inform practice. Saturation cannot truly be measured and is a subjective decision based solely on researcher judgment. It can be argued, therefore, that there was enough data from the interviews to ensure that the research questions were answered.

The nature of the research question and the interview questions need to be considered also. Research into sex and sexuality warrants its own set of limitations including but not limited to fearful and hesitant participants. Sexuality is considered a sensitive and private topic, which is fed into by social, cultural, moral, and legal norms and restraints, and may involve stigmatized and/or illegal behavior. This limits the number of individuals willing to speak of their sexuality with health professionals, which may explain the modest number of participants.

References:

1. Ulin, P. R., Robinson, E. T., & Tolley, E. E. (2005). *Qualitative methods in public health: A field guide for applied research* (1st ed.). San Francisco, CA: Jossey-Bass.
2. Fusch, P., & Ness, L. (2015). Are we there yet? Data saturation in qualitative research. *The Qualitative Report, 20*(9), 1408-1416.
3. Lee, D. T., Woo, J., & Mackenzie, A. E. (2002). The cultural context of adjusting to nursing home life: Chinese elders' perspectives. *Gerontologist, 42*(5), 667-675.
4. Baker, S., & Edwards, R. (2012). How many qualitative interviews is enough? Expert voices and early career reflections on sampling and cases in qualitative research. Southampton: National Centre for Research Methods.

We have added the following subsection after the **Procedure** subsection to address this issue.

> **Sample Size**
>
> The sample size for this qualitative study was eight carers, which is considered adequate for exploratory research in qualitative methodologies. In qualitative research, the focus is on in-depth understanding rather than statistical generalizability. The concept of "saturation" was used as a guide, defined as the point where additional data no longer contribute new insights to the research questions (Fusch & Ness, 2015; Ulin et al., 2005). According to qualitative research standards, a sample size of eight can be sufficient for generating meaningful insights, especially in studies involving sensitive topics like hypersexuality in PD. This number allows for a thorough exploration of individual experiences and contributes to theory development within the constraints of qualitative research.

Data Collection Method

The semi-structured interviews, lasting from two to four hours, may have caused participant fatigue, potentially affecting the quality and depth of responses. Long interviews can be exhausting, resulting in less detailed or consistent responses. How did the authors acknowledge and address this issue?

Response:

We anticipated the potential for participant fatigue due to the length of the semi-structured interviews. To minimize this impact, we employed several strategies. The interviewer was trained to monitor signs of fatigue and offered participants breaks as needed. Additionally, the interview structure allowed for flexibility, enabling participants to elaborate on topics they felt most comfortable discussing while avoiding unnecessary strain. We also reviewed transcripts to assess whether response quality declined over time, ensuring that key themes were consistently addressed throughout the interviews. These steps helped maintain the depth and reliability of the data collected.

Detailing of Thematic Analysis

While thematic analysis is an excellent approach, the article could have provided a more detailed description of the coding and categorisation process of the qualitative data. The lack of details on the analysis process may raise questions about the study's transparency and replicability. Including a more comprehensive description of the coding process, with examples of interview excerpts illustrating each theme, would enhance the transparency and reliability of the results.

Response:

Thank you for your comment. We acknowledge the importance of providing a more detailed description of the coding and categorization process of the qualitative data. Therefore, we have added the following paragraph under the **Data Analysis** section.

> Initially, interview transcripts were reviewed and organized into an Excel chart to facilitate data accessibility and ensure comprehensive analysis. This systematic arrangement allowed researchers to examine participant responses to each interview question without repeatedly referring to full transcripts. Following data

> familiarization, key extracts were identified through annotation and highlighting, capturing recurring words, ideas, and patterns. These extracts were systematically grouped into codes. Researchers then compared and refined codes through discussion, establishing coherent relationships and categorizing them into preliminary themes. Themes were subsequently reviewed for coherence, consistency, and distinctiveness. Based on this evaluation, themes were retained, modified, or removed as necessary. Subthemes were identified where applicable, representing distinct yet interconnected elements within overarching themes.

We also illustrated each theme with examples of interview excerpts. Initially, due to manuscript word limits, we placed these quotes in **Table S1**. However, recognizing the importance of including examples within the main text as highlighted by the COREQ checklist, we revised the manuscript accordingly.

Discussion of Limitations

The study's limitations section is adequately addressed but could be expanded to discuss methodological limitations and their implications more deeply. A more detailed discussion would help better understand the potential biases and limitations of the study, such as the influence of response biases and the limitation in generalising the results.

Response:

We appreciate the reviewer’s suggestion to expand the discussion of methodological limitations. In response, we have elaborated on potential response biases and limitations in generalizability. However, we believe that our study design, including in-depth qualitative methods and a focus on spousal carers, provides valuable insights that complement broader quantitative research. We have now clarified these points in the revised **Limitations** section:

> While this study focused on spousal carers, the impact of hypersexuality extends to other family members and professional carers, warranting broader investigation. The small sample size limited the generalizability of findings and restricted the ability to perform in-depth quantitative analyses. Future studies with larger and more diverse samples could better explore relationships between disease severity, medication effects, and hypersexuality, enhancing the applicability of results across different patient demographics and clinical settings.
>
> Additionally, qualitative research is inherently subject to response biases, such as social desirability bias, where participants may have underreported or framed their experiences in a way they perceived as socially acceptable. The sensitive nature of hypersexuality may have further influenced participants’ willingness to fully disclose their experiences. While we mitigated this by fostering a confidential and nonjudgmental interview environment, future research could incorporate anonymous surveys or mixed-method approaches to capture a broader range of perspectives.

In conclusion, while the article offers valuable insights into the impact of hypersexuality on caregivers, methodological improvements, sample expansion, and greater detail in the analysis and discussion would significantly strengthen the research. These enhancements would increase the practical applicability of the findings, contributing to better support for caregivers and patients affected by hypersexuality.

Reviewer: 2
Dr. Annelien Duits, Maastricht University Medical Centre+ Brain+Nerve Center, Radboudumc
Comments to the Author:
Hyperseksuality is a relevant topic especially with respect to caregiver burden. However, in my opinion, the overall impact of hypersexuality is already well-known, perhaps not at the level of the individual caregiver but certainly at that of family caregivers in general (e.g., stigma, frustration) and the quality of the relationship with the person with Parkinson's disease (PD) and dementia. The type of hypersexuality such as the more impulsive and compulsive type in PD versus disinhibition and inappropriateness in dementia (FTD in particular) has more news value and needs more attention in the discussion. In addition to a larger sample size or other methodological issues, the underlying mechanisms could also be addressed here.

Although there is little new information, the paper is well written and qualitative studies are very welcome to outline the impact of a problem.

Some suggestions for improvement of the present paper are:

Please explain why it was not possible to involve patients in designing the study.

Response:
Thank you for your comment. Under the **Patient and Public Involvement** section, we meant it as in they were not involved in the conceptualization of the study, not that they were not involved in the project. This study was actually part of a broader UCL project examining hypersexuality in neurological disorders, which includes a recently published systematic review (Tayim, Barbosa, et al., 2024) and a qualitative study exploring the clinical phenomenology and impact of hypersexuality in patients with Parkinson's Disease (Tayim, Panicker, et al., 2024). We added this information in the **Study Design** section. Nevertheless, it was not possible to include dementia patients because the research team was informed that dementia patients could not be approached about taking part in the study (Tayim, Panicker, et al., 2024) due to their research fatigue and cognitive decline.
Refer to the supplemental info about the carer assessment interview, now a reference in the text is lacking, while the interview has been added to the manuscript.

Response:
Thank you for your comment. We have referenced the carer assessment interview as **Supplementary Appendix 2** as the interview was developed by the authors and is not published anywhere.
Please ad a paragraph on rigour and reflexivity, see the Consolidated Criteria for Reporting Qualitative Research (COREQ) (Tong, Sainsbury, and Craig, 2007)

Response:
Thank you for your suggestion. We have added the following section at the end of the **Methods** section:

Rigor and Reflexivity

To ensure methodological rigor, we adhered to the Consolidated Criteria for Reporting Qualitative Research (COREQ) (Tong et al., 2007). Strategies to enhance trustworthiness included investigator triangulation, whereby multiple researchers participated in coding, theme generation, and data interpretation to minimize individual biases. Member checking was conducted informally, allowing participants to clarify or expand on their responses during interviews, ensuring the authenticity of the data. Reflexivity was maintained throughout the research process, with researchers critically examining their own preconceptions and potential influences on data collection and analysis. Regular discussions within the research team facilitated awareness of positionality and its impact on interpretation, thereby strengthening the credibility and dependability of the findings.

Consider figure 2 and 3 as part of the results instead of the discussion.

Response:
Thank you for your suggestion. Now we have included both figures under the **Results** section.

Discuss the small sample size with respect to the criteria of qualitative research (individual interviews) such as saturation, homogeneity of the sample etc. The present sample is relatively heterogenous given the different diagnoses. No information has been given about saturation and besides that saturation is a debatable issue. See for instance Vasileiou, K., Barnett, J., Thorpe, S. et al. 2018)

Response:
Thank you for your comment. One of the most debated issues in qualitative interviewing and analysis is whether it is possible, or even desirable, to estimate the number of interviews to conduct prior to undertaking them and the number of interviews required to reach "saturation". Saturation can be defined as the point researchers reach whereby more data would no longer be of benefit in answering the research questions (1, 2). A study by Lee et al. (2002) suggests that studies using more than one method requires fewer participants (3). Further, a paper by Baker and Edwards (2012) published by the National Centre for Research Methods reports a study that included 14 renowned social scientists and 5 early career researchers who were all asked the question "How many qualitative interviews is enough?" (4). The consensus was generally that "it depends on the purpose of the research" (4). The below table reports some of the answers:

"How many qualitative interviews is enough": Responses from paper by Baker and Edwards (2012)

Expert	Response
Adler and Adler (p. 8)	12-60 interviews (mean = 30)<br>Conducting too many interviews may not only be unnecessary and time consuming, but may also reduce the time given to the analysis of data consequently reducing the quality of the analysis
Becker (p. 15)	One interview may be enough to give a rich account of a unique event<br>A few may be enough if the purpose is to highlight that an issue is more complex than previously though
Bryman (p. 18)	>20 interviews for purely interview-based studies

Charmaz (p. 21)<br>and Doucet (p. 25)	Depends on career stage of researcher, their analytical ambitions, and the community reviewing the study
Jenson (p. 39)	Depth and complexity of analysis matter more than sample size
Mason (p. 29)	Cautions against "knee-jerk reaction" that more interviews are always better

When compared to quantitative studies, yes - the sample size presented is small (relatively); however, for qualitative research, it is sufficient enough to generate theory and inform practice. Saturation cannot truly be measured and is a subjective decision based solely on researcher judgment. It can be argued, therefore, that there was enough data from the interviews to ensure that the research questions were answered.

The nature of the research question and the interview questions need to be considered also. Research into sex and sexuality warrants its own set of limitations including but not limited to fearful and hesitant participants. Sexuality is considered a sensitive and private topic, which is fed into by social, cultural, moral, and legal norms and restraints, and may involve stigmatized and/or illegal behavior. This limits the number of individuals willing to speak of their sexuality with health professionals, which may explain the modest number of participants.

References:

1. Ulin, P. R., Robinson, E. T., & Tolley, E. E. (2005). *Qualitative methods in public health: A field guide for applied research* (1st ed.). San Francisco, CA: Jossey-Bass.
2. Fusch, P., & Ness, L. (2015). Are we there yet? Data saturation in qualitative research. *The Qualitative Report, 20*(9), 1408-1416.
3. Lee, D. T., Woo, J., & Mackenzie, A. E. (2002). The cultural context of adjusting to nursing home life: Chinese elders' perspectives. *Gerontologist, 42*(5), 667-675.
4. Baker, S., & Edwards, R. (2012). How many qualitative interviews is enough? Expert voices and early career reflections on sampling and cases in qualitative research. Southampton: National Centre for Research Methods.

We have added the following subsection after the **Procedure** subsection to address this issue.

Sample Size

The sample size for this qualitative study was eight carers, which is considered adequate for exploratory research in qualitative methodologies. In qualitative research, the focus is on in-depth understanding rather than statistical generalizability. The concept of "saturation" was used as a guide, defined as the point where additional data no longer contribute new insights to the research questions (Fusch & Ness, 2015; Ulin et al., 2005). According to qualitative research standards, a sample size of eight can be sufficient for generating meaningful insights, especially in studies involving sensitive topics like hypersexuality in PD. This number allows for a thorough exploration of individual experiences and contributes to theory development within the constraints of qualitative research.

Finally discuss the added value (unless limitations) of the present findings. Are the findings helpful in designing treatments other than education?

Response:
Thank you for your comment. We have amended the **Implications** section as follows:

> **Implications**
> This study highlights the critical need for healthcare professionals to educate patients and carers about ICDs associated with PD and dementia, including hypersexuality, and to provide ongoing support and monitoring (R. De Giorgi & H. Series, 2016; Weintraub et al., 2009). Targeted psychological and behavioral strategies could help carers manage distress and improve coping mechanisms. Acceptance and commitment therapy (ACT) (Hayes et al., 2006) may be particularly beneficial, as it encourages carers to accept the challenges of their partners' hypersexual behaviors while fostering psychological flexibility and values-based action. Group-based interventions, such as structured peer-support programs modeled after Al-Anon (Kverme, 1990), could provide a shared space for carers to exchange experiences, reduce isolation, and develop practical coping strategies. Additionally, cognitive-behavioral therapy (CBT) tailored for carers could address maladaptive thought patterns and emotional distress related to managing hypersexual behaviors. Psychosocial interventions, including couple-based therapy and family counseling, may also facilitate communication and adaptive strategies.

VERSION 2 - REVIEW

**Reviewer**	**2**
**Name**	**Duits, Annelien**
**Affiliation**	**Maastricht University Medical Centre+ Brain+Nerve Center**
**Date**	**18-Mar-2025**
**COI**	

Thanks for the careful revision and feedback on the comments.

See below for some (minor) suggestions.

Strengths and limitations are overlapping and the heterogeneity and sample size could be included as a limitation?

Study objective: please specify dementia to FTD.

This study was conducted from April 2015 to August 2017. It was part of a broader UCL project examining hypersexuality in neurological disorders … mentioning the references is sufficient given the word count.

In the procedure it was mentioned that eight carers were successfully recruited

into the study. Later this number was supposed to be based on saturation? Was it? As it seems to be a retrospective conclusion rather than a strategy prior to recruitment. Maybe it is better to elaborate on this (sample size and saturation) in the discussion.

According to qualitative research standards, a sample size of

eight can be sufficient for generating meaningful insights, especially in studies involving sensitive topics like hypersexuality in PD. Is there a reference?

The small sample size limited the generalizability of findings and

restricted the ability to perform in-depth quantitative analyses. Please explain? This was not intended. But of course, a mixed method is of additional value. I would suggest limiting the 'limitations' to this qualitative study only and mention the added value of a mixed method with quantitative analyses in future research.

VERSION 2 - AUTHOR RESPONSE

Reviewer: 2
Dr. Annelien Duits, Maastricht University Medical Centre+ Brain+Nerve Center, Radboudumc
Comments to the Author:
Thanks for the careful revision and feedback on the comments.
See below for some (minor) suggestions.

Strengths and limitations are overlapping and the heterogeneity and sample size could be included as a limitation?

Response:
Thank you for your comment. We included a **Strengths and limitations of this study** section in response to the following editor's comment in the first revision: "Please add a section entitled 'Strengths and limitations of this study', immediately after the abstract." We have discussed issues surrounding the sample size in our responses to your below comments.
Study objective: please specify dementia to FTD.

Response:
Thank you for your comment. The objective of our study was to explore the impact of hypersexuality on spousal carers of patients with PD and dementia, including both Alzheimer's disease (AD) and frontotemporal dementia (FTD). However, despite our initial aim to include carers of patients with AD, we were only able to successfully recruit carers of patients with FTD. We acknowledge this and have clarified this point in the **Limitations** section of our manuscript.
This study was conducted from April 2015 to August 2017. It was part of a broader UCL project examining hypersexuality in neurological disorders … mentioning the references is sufficient given the word count.

Response:
Thank you for your suggestion. We have shortened the sentence accordingly.
In the procedure it was mentioned that eight carers were successfully recruited into the study. Later this number was supposed to be based on saturation? Was it? As it seems to be a retrospective conclusion rather than a strategy prior to recruitment. Maybe it is better to elaborate on this (sample size and saturation) in the discussion.

Response:
Thank you for your comment. Under the **Sample Size** section, we had mentioned that "The concept of "saturation" was used as a guide." In other words, we had the concept of "saturation" in mind during the recruitment process. We understood that "saturation" is not a number per se, but as Fusch and Ness (2015) put it:

> "there is no one-size-fits-all method to reach data saturation; moreover, more is not necessarily better than less and vice versa. […] When deciding on a study design, the student should aim for one that is explicit regarding how data saturation is reached. Data saturation is reached when there is enough information to replicate the study (O'Reilly & Parker, 2012; Walker, 2012), when the ability to obtain additional new information has been attained (Guest et al., 2006), and when further coding is no longer feasible (Guest et al., 2006). Rich and thick data descriptions obtained through relevant data collection methods can go a long ways towards assisting with this process when coupled with an appropriate research study design that has the best opportunity to answer the research question."

Simultaneously, we reached a point where we could not recruit more than eight carers, and Guest et al. (2006) have argued that "basic elements for metathemes were present as early as six interviews," so we proceeded with those eight. We have amended the **Sample Size** section accordingly:

> **Sample Size**
> The sample for this qualitative study comprised eight carers, a size considered sufficient for exploratory research within qualitative methodologies. Qualitative research prioritizes in-depth understanding over statistical generalizability, with sample size determined by the principle of thematic saturation. Saturation, in this context, refers to the point where additional data collection yields no new insights relevant to the research questions [14, 15]. This approach aligns with Fusch and Ness (2015), who emphasize that "more is not necessarily better than less," challenging the notion of a fixed target number for saturation [15]. Instead, saturation is reached when the data adequately represent the phenomenon under study, enable study replication, and further coding produces redundant information. Moreover, Guest et al. (2006) posit that a sample of six can generate "basic elements for metathemes", especially in studies involving sensitive topics [16]. Consequently, the data obtained from eight carers allowed for a thorough exploration of individual experiences, contributing to theory development within the inherent constraints of qualitative research.

Since this aspect is comprehensively described in the methods, its inclusion in the discussion would not yield novel information.

According to qualitative research standards, a sample size of eight can be sufficient for generating meaningful insights, especially in studies involving sensitive topics like hypersexuality in PD. Is there a reference?

Response:
We addressed this point in the above response (we also changed certain phrasings).

The small sample size limited the generalizability of findings and restricted the ability to perform in-depth quantitative analyses. Please explain? This was not intended. But of course, a mixed method is of additional value. I would suggest limiting the 'limitations' to this qualitative study only and mention the added value of a mixed method with quantitative analyses in future research.

Response:
Thank you for your comment. We amended the **Limitations** section in the following way:

> **Limitations**
> This study encountered several limitations. Firstly, while the sample included carers of patients with PD and FTD, the intended inclusion of carers of patients with AD was not realized. This restricted our ability to compare the impact of hypersexuality across dementia subtypes, specifically AD. Future research should prioritize recruiting a diverse sample, including carers of patients with AD, to achieve a more comprehensive understanding. Secondly, the study's focus on spousal carers limited the scope of investigation. The impact of hypersexuality extends to other family members and professional carers, warranting broader investigation. Thirdly, inherent to qualitative research, response biases, such as social desirability, may have influenced participant disclosures, particularly given the sensitive nature of hypersexuality. Although a confidential and nonjudgmental interview environment was established, future studies could consider incorporating anonymous surveys or mixed-methods designs to mitigate this potential bias. Finally, while this qualitative approach yielded rich, in-depth insights, a mixed-methods design, integrating quantitative analyses, would provide greater triangulation of findings and enhance the robustness of conclusions, offering a more complete understanding of the phenomenon.
